# Nanotechnology for ischemic stroke treatment: addressing challenges across stroke management stages

**DOI:** 10.3389/fmolb.2026.1794828

**Published:** 2026-05-12

**Authors:** Yue Wu, Lijun Wang, Chen Zou, Xianfu Meng, Pengfei Yang, Hongjian Zhang

**Affiliations:** 1 School of Health Science and Engineering, University of Shanghai for Science and Technology, Shanghai, China; 2 Oriental Pan-Vascular Devices Innovation College, University of Shanghai for Science and Technology, Shanghai, China; 3 Neurovascular Center, Changhai Hospital, Naval Medical University, Shanghai, China; 4 Department of Nuclear Medicine, Shanghai Key Laboratory of Nautical Medicine and Translation of Drugs and Medical Devices, Changhai Hospital, Naval Medical University, Shanghai, China; 5 Department of Materials Science, State Key Laboratory of Molecular Engineering of Polymers, Fudan University, Shanghai, China; 6 State Key Laboratory of Neurology and Oncology Drug Development, Nanjing, China

**Keywords:** cerebral ischemia, ischemic stroke, nanomedicines for ischemic stroke treatment, staged intervention, stroke management stage

## Abstract

Ischemic stroke is a leading cause of mortality and long-term disability, with single acute interventions such as thrombolysis and mechanical thrombectomy limited by narrow therapeutic windows, hemorrhage risks, and challenges in crossing the blood-brain barrier (BBB). This review focuses on targeted therapeutic strategies across the four key stages of ischemic stroke management: prevention, neuroprotection, revascularization, and adjunctive therapy. By systematically analyzing each stage of ischemic stroke, we emphasize the necessity of staged intervention and the essential role of nanomedicines during each stage. From controlled drug release in the prevention stage to the extension of therapeutic time windows for acute intervention and to enhancing adjunctive outcomes in later stages, nanomedicines provide solutions to optimize treatment efficacy and minimize side effects. Finally, we provide perspectives on spatiotemporal control in nanomedicine for precision stroke treatment, including blood pressure and infarct temperature regulation, the crosstalk between systems, and gene therapy. This review underscores the transformative role of nanomedicines in ischemic stroke treatment and the potential to revolutionize stroke management by overcoming current clinical barriers and improving patient outcomes.

## Graphical Abstract

**Figure:**
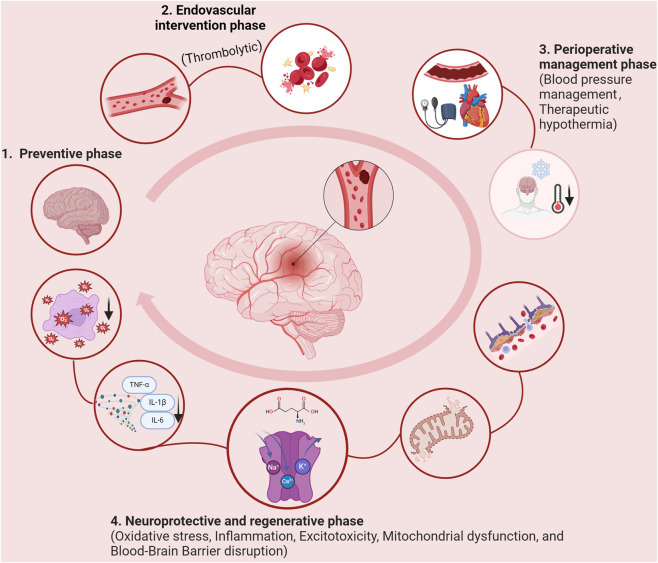

## 1 Introduction

Stroke remains a leading cause of death and long-term disability worldwide, comprising two major subtypes: ischemic and hemorrhagic. Of these, ischemic stroke is the predominant form, accounting for approximately 65% of cases globally (Feigin et al., 2025). In the United States, nearly 800,000 individuals experience a stroke each year, corresponding to one stroke approximately every 40 s, and stroke-related deaths occur about every 3 min (Waqas et al., 2025). Ischemic stroke is caused by the obstruction of cerebral blood vessels, resulting in reduced cerebral blood flow (CBF) and subsequent neuronal injury. Despite significant advancements in elucidating the pathophysiology of ischemic stroke, effective therapeutic options remain scant. Interventions such as intravenous thrombolysis, mechanical thrombectomy (MT), and neuroprotective agents have demonstrated limited efficacy in mitigating cerebral injury (Powers, 2020; Chamorro et al., 2016; Huguet et al., 2016). These limitations are mainly attributed to the narrow therapeutic window, the complex brain microenvironment (e.g., hemorrhagic risk), modest treatment efficacy, and the poor ability of drugs to cross the blood-brain barrier (BBB) (Liao et al., 2022).

**FIGURE 1:**
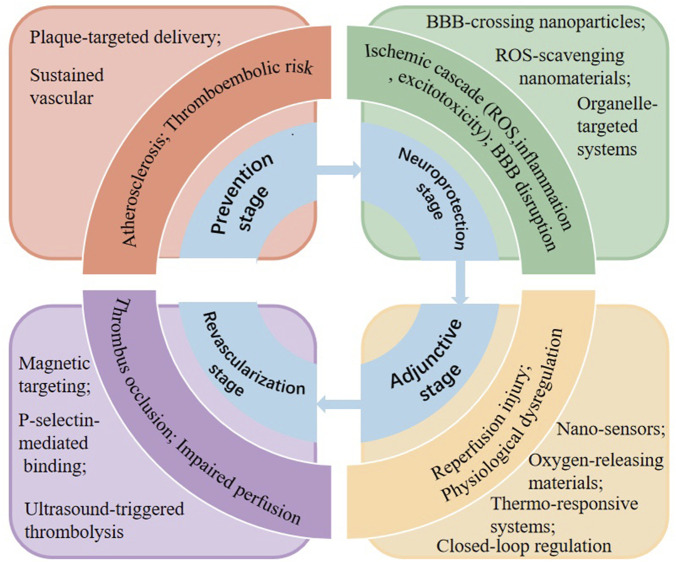
Stage-based management of ischemic stroke: pathological challenges and nanotechnology-enabled spatiotemporal control. The schematic summarizes the four stages of ischemic stroke-prevention, neuroprotection, revascularization, and adjunctive stage-and the corresponding pathological mechanisms and nanotechnology-based therapeutic strategies. Nanomedicine enables targeted delivery, controlled release, thrombus-specific intervention, and real-time physiological regulation, thereby achieving spatiotemporal control across stroke progression.

In clinical practice, the management of ischemic stroke has been traditionally focused on the revascularization stage, utilizing interventions like intravenous thrombolysis and MT to rapidly restore blood flow. However, this narrow concentration frequently neglects the comprehensive spectrum of care necessary for optimal stroke management. Effective ischemic stroke management should instead be viewed as a continuous and stage-adapted process that addresses the evolving pathological mechanisms throughout stroke progression. Within this perspective, therapeutic strategies extend beyond reperfusion and involve preventive, neuroprotective, and supportive interventions targeting different phases of disease development. By considering the temporal evolution of ischemic injury, clinicians can more effectively target key pathological mechanisms and improve overall treatment outcomes. The overall stage-based framework and corresponding nanotechnology-enabled strategies are schematically illustrated in Figure 1.

**FIGURE 2:**
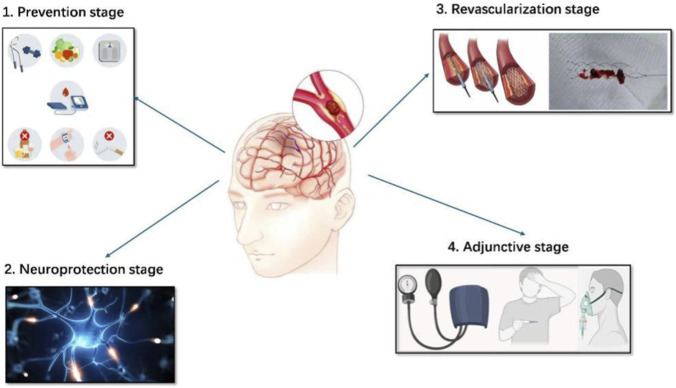
Schematic representation of the four key stages of ischemic stroke management: prevention stage, neuroprotection stage, revascularization stage, and adjunctive stage.

Therapeutic management of ischemic stroke therefore involves several intervention contexts—including prevention, neuroprotection, revascularization, and adjunctive care—each associated with distinct pathological mechanisms and clinical challenges (Figure 2).

During the prevention stage, the emphasis is placed on modulating risk factors—particularly hypertension and hyperlipidemia—through pharmacological interventions such as angiotensin-converting enzyme (ACE) inhibitors, angiotensin receptor blockers (ARBs), and statins (e.g., atorvastatin). Additionally, anticoagulants like warfarin and novel oral anticoagulants (NOACs; dabigatran, rivaroxaban, apixaban) are administered to prevent thromboembolic events. However, these regimens are limited by side effects, including bleeding risks associated with anticoagulants and suboptimal control of blood pressure or lipid levels (Herpich and Rincon, 2020; Liao et al., 2022).

The neuroprotection stage aims to minimize ischemic injury and extend the therapeutic window by mitigating processes such as oxidative stress, inflammation, and excitotoxicity. Agents like antioxidants (e.g., edaravone) and calcium channel blockers (e.g., nimodipine) have been employed for this purpose. Yet, their efficacy remains limited due to poor permeability across the BBB and the lack of precise molecular targeting (Lakhan et al., 2009).

In the revascularization stage, therapeutic focus shifts to the rapid restoration of CBF. Intravenous thrombolysis with alteplase is a widely used strategy but is constrained by a narrow therapeutic window of 4.5 h post-symptom onset. MT provides an alternative for large vessel occlusion, though its success is often hindered by technical difficulties in accessing distal or tortuous thrombus locations (Brott and Bogousslavsky, 2000; Kirkman et al., 2014)

The adjunctive stage aims to consolidate treatment efficacy and promote neurological recovery following revascularization. Therapeutic hypothermia (TH) is widely explored for its ability to reduce metabolic demand and mitigate reperfusion injury, but conventional cooling methods often lack brain specificity and are associated with complications such as infection, shivering, and coagulopathy (Kalil and Branecki, 2014). Oxygen therapy seeks to alleviate hypoxia but remains controversial, as excessive oxygen may exacerbate oxidative stress (Nakane, 2020). Additionally, neuroregenerative support using growth factors or stem cell-derived agents holds promise but is limited by poor stability, short half-life, and low BBB permeability (Gupta and Singh, 2022).

Nanotechnology has emerged as a promising tool in ischemic stroke treatment, offering distinct advantages over conventional therapies (Lin et al., 2022). Specifically, in the prevention stage, conventional antihypertensive and anticoagulant drugs often cause systemic side effects. Nanoparticle-based delivery systems enable sustained and targeted drug release, improving safety and therapeutic efficiency (Ingalls, 2019). During the neuroprotection stage, conventional agents such as antioxidants and anti-inflammatory drugs show limited efficacy due to poor BBB permeability and lack of targeting. Nanoparticles can cross the BBB and accumulate in ischemic regions, enhancing neuroprotection by reducing oxidative stress and inflammation (Wang et al., 2024b; Li et al., 2024b; Fayyazi et al., 2023). In the revascularization stage, thrombolytic therapy with agents like alteplase is restricted by a short time window and bleeding risk. Nanomedicines enable targeted thrombolysis, lowering dosage and hemorrhagic complications while potentially extending the treatment window (Ray, 2019). Finally, in the adjunctive stage, traditional supportive measures often lack precision and adaptability. In contrast, nanotechnology enables real-time monitoring and dynamic regulation of physiological parameters such as temperature and oxygenation, offering improved safety and better support for neurological recovery (Varadan, 2010).

A critical advantage of nanomedicine over conventional pharmacotherapy lies in its ability to achieve spatiotemporal control of therapeutic interventions. Traditional drugs are typically distributed systemically and act continuously once administered, offering limited control over where and when therapeutic effects occur. In contrast, nanotechnology enables precise regulation of therapeutic action across both spatial and temporal dimensions. Spatially, nanocarriers can be engineered to selectively accumulate in thrombi, ischemic brain regions, specific cell types, or even subcellular organelles such as mitochondria. Temporally, stimuli-responsive or controlled-release nanoplatforms allow drugs to be delivered at defined stages of disease progression, synchronized with the evolving pathophysiology of ischemic stroke.

This capability is particularly important in ischemic stroke management, where the dominant pathological mechanisms change dynamically from the prevention stage to neuroprotection, revascularization, and post-reperfusion recovery. Therefore, understanding how nanotechnology enables stage-specific and spatiotemporally controlled intervention provides a unifying framework for evaluating nanomedicine strategies in ischemic stroke.

## 2 Four key stages for ischemic stroke

Current ischemic stroke management has largely focused on the revascularization stage, aiming to restore cerebral blood flow via thrombolysis or MT. However, this approach alone is insufficient for comprehensive care. Optimal ischemic stroke management should span four key stages, including prevention, neuroprotection, revascularization, and adjunctive stage, each of which can address distinct pathological processes. Importantly, these stages should be viewed as interconnected therapeutic windows within a dynamically evolving disease process rather than isolated treatment phases.

In the prevention stage, the focus lies in managing modifiable risk factors such as hyperlipidemia and thrombosis through lipid-lowering agents, antiplatelet drugs, and anticoagulants (Williams and Felix, 2022). The neuroprotection stage targets early brain injury through four key strategies: reducing oxidative stress, mitigating excitotoxicity, suppressing inflammation, and preserving as well as restoring BBB integrity (Lehtonen and Jolkkonen, 2024). The revascularization stage emphasizes timely reperfusion to minimize infarct volume and improve prognosis (Ciccone et al., 2013). Finally, the adjunctive stage facilitates recovery by employing strategies such as oxygen therapy and therapeutic hypothermia to minimize secondary damage and support rehabilitation (Lim et al., 2024). This stage-based framework enables more targeted interventions and offers a comprehensive strategy to improve stroke outcomes. In this review, the neuroprotection stage primarily refers to interventions targeting the intrinsic ischemic injury cascade within brain tissue, such as oxidative stress, excitotoxicity, inflammation, mitochondrial dysfunction, and BBB disruption, with the goal of preserving the ischemic penumbra and extending the therapeutic window for reperfusion therapies. In contrast, the adjunctive stage is defined as the post-revascularization period that focuses on stabilizing systemic and cerebral physiological parameters—such as blood pressure, temperature, and oxygenation—to mitigate secondary injury and support neurological recovery.

While nanoparticle-based preventive strategies improve pharmacokinetic stability and targeting precision compared with conventional drugs, their long-term clinical value will depend on factors such as biosafety during chronic administration, scalability of manufacturing, and patient adherence, which remain insufficiently addressed in most current preclinical studies.

### 2.1 Prevention stage

**FIGURE 3:**
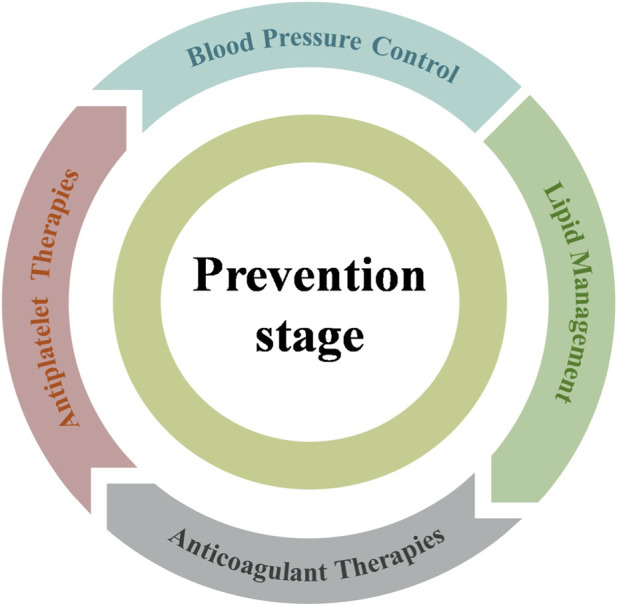
Nanotherapeutics in prevention stage of ischemic stroke.

Prevention constitutes a critical component of ischemic stroke management. It can prevent the initial onset of ischemic stroke and significantly reduce the healthcare burden associated with long-term disability and recurrent events. Prevention of ischemic stroke involves the management and mitigation of established risk factors, such as blood pressure regulation, lipid modulation, antiplatelet and anticoagulant therapies, (Dawson et al., 2022; Sagris et al., 2024), which are major contributors to ischemic stroke incidence (Kernan et al., 2014). In light of these challenges, this section will discuss both traditional treatments and nanoparticle-based strategies aimed at improving stroke prevention outcomes (Figure 3).

#### 2.1.1 Blood pressure control

**FIGURE 4:**
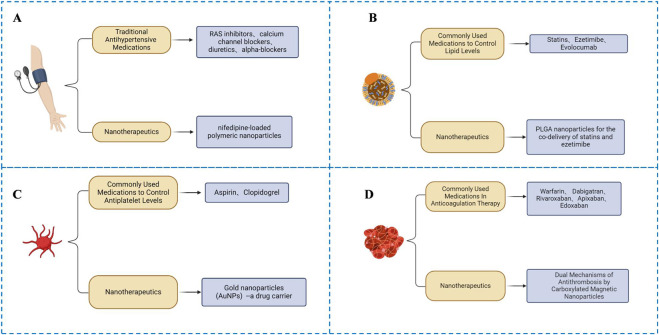
Comprehensive management strategies for ischemic stroke prevention, including **(A)** Hypertension management, **(B)** Lipid management, **(C)** Platelet management, and **(D)** Anticoagulation therapy.

Hypertension is the most prevalent and modifiable risk factor for ischemic stroke, accounting for over 50% of all cases due to its direct impact on vascular integrity and hemodynamic stress (Goldstein et al., 2001; Webb and Werring, 2022). Epidemiological evidence suggests that a 5 mmHg reduction in systolic blood pressure (SBP) can reduce the risk of stroke by approximately 10%, reinforcing the clinical importance of tight blood pressure control (Blood Pressure Lowering Treatment Trialists’ Collaboration, 2021). Commonly prescribed antihypertensive agents include renin-angiotensin system (RAS) inhibitors (e.g., ACE inhibitors, ARBs), beta-blockers, calcium channel blockers, and diuretics (Goyal et al., 2021; Karagiannaki et al., 2024; Lakatos et al., 2024; Bawiskar et al., 2023; Cressman and Gifford, 1983) (Figure 4A). However, traditional antihypertensive therapies face challenges such as variable pharmacokinetics, which can lead to unstable plasma drug concentrations and fluctuations in blood pressure. This is especially problematic with short-acting calcium channel blockers like nifedipine, which can induce rapid blood pressure drops, cerebral hypoperfusion, and reflex tachycardia, thereby increasing the risk of ischemic events in susceptible patients (Hsu et al., 2019; Vidt, 2001; Furberg et al., 1995).

To overcome these limitations, nanoparticle-based drug delivery systems have been developed to improve the pharmacokinetic profile, stability, and bioavailability of anti-hypertensive agents. These systems enable sustained and controlled drug release, offering more stable blood pressure control and reduced risk of adverse hemodynamic changes. One notable early approach was introduced by Kim et al. (1997), who designed nifedipine-loaded polymeric nanoparticles using biodegradable materials such as poly (ε-caprolactone) (PCL), polylactic-co-glycolic acid (PLAGA), and Eudragit RL/RS (Kim et al., 1997). These nanoparticles were engineered to prolong the release of nifedipine, decrease peak plasma concentrations, and minimize the risk of excessive vasodilation. Pharmacokinetic studies demonstrated that nanoparticle formulations significantly extended the mean residence time (MRT) of nifedipine, ensuring a smoother and more sustained antihypertensive effect over time compared to conventional polyethylene glycol (PEG)-based formulations.

The controlled release of nifedipine from these nanoparticles operates primarily via a diffusion-regulated mechanism, which helps maintain steady plasma drug levels and avoids sudden spikes that can lead to rapid hypotension or reflex tachycardia.

Building upon this foundation, Tagliari et al. (2015) formulated advanced nifedipine nanocapsules using Pluronic F68 and polyvinyl alcohol (PVA) as stabilizers (Tagliari et al., 2015). These modifications aimed to enhance drug loading capacity, photostability, and controlled release kinetics. Pluronic F68-based nanocapsules exhibited superior drug encapsulation and maintained prolonged plasma concentrations, while PVA-stabilized nanocapsules demonstrated a faster onset of action due to more rapid drug release. Both systems showed improved antihypertensive efficacy and bioavailability in hypertensive rat models, effectively minimizing initial blood pressure drops and sustaining long-term control.

Moreover, these nanoformulations reduced blood pressure variability and protected against oxidative stress and endothelial damage, two key contributors to stroke pathogenesis. Overall, nanoparticle-based delivery of antihypertensive agents represents a promising strategy to achieve more consistent blood pressure regulation, enhance drug performance, and reduce the risk of ischemic stroke in high-risk populations.

Nevertheless, blood pressure regulation in stroke prevention is governed by complex physiological feedback systems, suggesting that improvements in drug delivery alone may not fully substitute for adaptive hemodynamic regulation.

#### 2.1.2 Lipid management

Elevated blood lipid levels promote atherosclerosis, the primary pathological basis of ischemic stroke (Gofman and Lindgren, 1950). Acute arterial occlusion caused by plaque rupture or thrombosis on these lesions is a major trigger of ischemic events (Langer, 1991). Among blood lipids, low-density lipoprotein cholesterol (LDL-C) is most strongly associated with stroke risk, with each 1 mmol/L increase linked to a significantly higher incidence of ischemic stroke (Sun et al., 2019). Accordingly, lipid-lowering therapies play a central role in prevention strategies.

Statins are the most commonly prescribed agents, well recognized for their ability to reduce LDL-C levels and lower the risk of vascular events (Khan and Krasuski, 2020) (Figure 4B). Ezetimibe, which inhibits cholesterol absorption in the intestine, serves as an effective adjunct to statin therapy and further contributes to stroke risk reduction. Proprotein convertase subtilisin/kexin type 9 (PCSK9) inhibitors such as evolocumab provide additional LDL-C lowering when used in combination with statins. Accordingly, monotherapy often proves insufficient, and combination regimens are increasingly favored in clinical practice.

A randomized controlled trial showed that combining ezetimibe with moderate-intensity statins was more effective than high-intensity statins alone in patients with recent ischemic stroke (Hong et al., 2023). This combination achieved a ≥50% reduction in LDL-C and enabled more patients to reach target levels (<90 mg/dL) within 70 days, along with a lower incidence of major vascular events. Despite these advantages, ezetimibe suffers from variable bioavailability, often necessitating higher statin doses that increase the risk of myopathy and liver toxicity (Lv et al., 2024; Khan and Krasuski, 2020). In addition, the poor solubility and rapid metabolism of both drugs can result in inconsistent therapeutic outcomes, limiting the stability of long-term lipid control (Khan and Krasuski, 2020).

To address these limitations, Metwally et al. developed PLGA-based nanoparticles co-loaded with rosuvastatin and ezetimibe using an emulsion/solvent evaporation method (Metwally et al., 2024). These nanoparticles improved drug dissolution and enabled sustained release, leading to a threefold increase in rosuvastatin bioavailability and a twofold increase for ezetimibe *in vivo*. However, the systemic distribution of PLGA nanoparticles may lead to off-target effects and uncontrolled metabolism, thereby limiting their specificity for hepatic cholesterol regulation (Ganesh et al., 2015).

To improve targeting precision, Vanova Nakjinova et al. introduced lipid–polymer hybrid nanoparticles (LPHNPs), consisting of a polymeric core and a lipid–PEG shell (Vanova Nakjinova et al., 2022). This design enhanced nanoparticle stability and allowed preferential accumulation in the liver. By optimizing the ratio between hydrophilic rosuvastatin and lipophilic ezetimibe, the formulation achieved improved drug retention and controlled release. Compared to PLGA systems, LPHNPs demonstrated superior hepatic targeting, reduced systemic exposure, and improved safety profiles.

Beyond lipid regulation, LPHNPs may also exert anti-inflammatory effects. Elevated LDL-C levels are known to activate inflammatory pathways such as NF-κB and the NLRP3 inflammasome, leading to the release of pro-inflammatory cytokines like IL-1β and TNF-α (Bissonnette et al., 2023). These mechanisms contribute to atherosclerosis progression and post-stroke inflammation. By effectively lowering LDL-C and suppressing inflammatory signaling, LPHNPs may help alleviate vascular damage and reduce the inflammatory burden associated with ischemic stroke.

#### 2.1.3 Antiplatelet therapy

Abnormally elevated platelet counts or overactive platelet function increase blood viscosity and thrombosis risk, contributing to vascular events such as ischemic stroke and myocardial infarction (Mudhhi et al., 2024). Targeting the underlying mechanisms of thrombosis—including oxidative stress, inflammation, and endothelial dysfunction—is essential for stroke prevention. These factors impair endothelial integrity, activate platelets, and promote thrombus formation, creating a feedforward cycle of vascular injury and neuronal damage (Li et al., 2022b). Platelet aggregation further worsens ischemic injury by obstructing blood flow and exacerbating oxidative and inflammatory responses. These processes can disrupt the BBB, increasing the permeability to harmful molecules and immune cells, thereby aggravating secondary brain damage (Matthay et al., 2022).

Antiplatelet therapy plays a key role in interrupting this pathological cycle. Aspirin, as a first-line agent, inhibits platelet aggregation and reduces the risk of first-ever stroke (Johnston et al., 2020) (Figure 4C). However, its use in primary prevention must be carefully balanced against bleeding risk, and recent studies suggest that its benefits in some populations may be modest (Cloud et al., 2023). Therefore, the utilization of aspirin should be tailored to individual risk profiles and specific circumstances. Although combination therapy with multiple antiplatelet agents has shown effectiveness, it is accompanied by an augmented risk of bleeding (Johnston et al., 2020).

To address the limitations of conventional therapies, researchers have investigated nanomaterials with biological enzyme-like activity that influence platelet function through redox-related pathways (Manoharan et al., 2024). Ajdari et al. studied gold nanoparticles (AuNPs) of different sizes and their effects on platelet behavior and coagulation (Ajdari et al., 2017). Ajdari et al. synthesized AuNPs of various sizes and found that their effects on coagulation were highly size-dependent. Intermediate-sized particles, such as 45 nm AuNPs, significantly accelerated clot formation, while smaller particles (12 nm) had minimal effects, and certain larger particles (e.g., 85 nm) altered clot strength. This nonlinear trend suggests that particle size influences catalytic efficiency, possibly through differences in surface energy, protein corona formation, and platelet interaction dynamics. Transmission electron microscopy (TEM) imaging confirmed the spherical morphology of the particles. Due to altered surface characteristics, other morphologies such as nanorods or hollow spheres may exhibit different platelet-modulating behaviors and warrant further investigation. Platelet aggregometry and thromboelastography (TEG) assays further showed that AuNPs modulate platelet activity not by degrading platelets, but through surface interactions and catalytic effects, likely involving redox-mediated changes in coagulation pathways.

In a complementary approach, Guo et al. developed cell membrane-coated nanoparticles (CM-NPs) designed to reverse the effects of antiplatelet drugs such as ticagrelor and clopidogrel, which, despite their effectiveness in stroke prevention, increase the risk of bleeding (Guo et al., 2024). They genetically engineered 293T cells to overexpress P2Y receptors and harvested their membranes to coat mPEG-PLGA nanoparticles, forming biomimetic CM-NPs. These nanoparticles act as decoys by competitively binding to circulating P2Y_12_ inhibitors, thereby restoring platelet function without interfering with physiological clotting. The therapeutic mechanism of CM-NPs relies on surface-displayed P2Y_12_ receptors, which sequester free antiplatelet agents and prevent them from inhibiting native platelets. *In vitro* aggregation assays demonstrated that CM-NPs effectively reversed drug-induced platelet inhibition, while *in vivo* studies showed reduced bleeding time in mice treated with CM-NPs compared to control groups. Pharmacokinetic analysis further confirmed that CM-NPs accelerated the clearance of ticagrelor, supporting their utility as a rapid and targeted reversal agent.

Together, these two studies highlight distinct but complementary strategies for platelet modulation using nanotechnology. Ajdari et al.’s AuNPs offer a tunable platform for influencing coagulation dynamics, while Guo et al.’s CM-NPs provide an on-demand mechanism to neutralize antiplatelet drugs and mitigate bleeding risks. The integration of these approaches may contribute to more flexible and personalized strategies in ischemic stroke prevention and management.

Importantly, nanoparticle-mediated modulation of platelet activity must carefully balance thrombosis prevention with the risk of hemorrhage, particularly in patients with fragile cerebrovascular structures, highlighting the importance of tunable and reversible nanotherapeutic designs.

#### 2.1.4 Anticoagulant therapy

Oxidative stress and inflammation are key pathological processes in ischemic stroke and significantly contribute to endothelial dysfunction (Li et al., 2022b). Damage to endothelial cells promotes platelet adhesion and activation, initiating the coagulation cascade. This sequence ultimately leads to thrombus formation, vascular occlusion, reduced cerebral oxygenation, and neuronal injury (Lee et al., 2021; De Meyer et al., 2016). In patients with atrial fibrillation, blood stasis within the atria further elevates the risk of clot formation and subsequent cerebral embolism (Qureshi et al., 2022).

Anticoagulation therapy remains a cornerstone for stroke prevention by interrupting the coagulation cascade (Martin et al., 2021). It is especially beneficial in atrial fibrillation-related stroke prevention. Commonly prescribed agents for elderly patients include vitamin K antagonists (VKAs) such as warfarin and NOACs like dabigatran, rivaroxaban, apixaban, and edoxaban (Schäfer et al., 2020; Pandya, 2020). These drugs prevent thrombus formation via different mechanisms of coagulation regulation (Figure 4D).

Despite their clinical value, conventional anticoagulants present notable drawbacks. Warfarin requires frequent monitoring and is susceptible to dietary and drug interactions, while NOACs, although more convenient, still carry systemic bleeding risks due to their non-targeted distribution (Skirdenko and Nikolaev, 2021; Tarar et al., 2021). Moreover, neither VKAs nor NOACs effectively dissolve existing thrombi, limiting their application in patients with established thrombosis (Nutescu et al., 2004).

Magnetic nanoparticles (MNPs) offer a promising alternative. They can be magnetically directed to thrombi and exhibit enhanced thrombolytic activity when exposed to oscillating magnetic fields (Zhang and Jiang, 2023; Chen et al., 2019). This enables localized drug accumulation, magnetically responsive release, and reduced systemic side effects. Additionally, MNPs generate mechanical forces under magnetic stimulation, disrupting the fibrin meshwork and enhancing thrombolysis.

Bian et al. developed HOOC-PEG2000-coated Fe_3_O_4_ MNPs using a thermal decomposition method (Bian et al., 2023). These nanoparticles function through two main mechanisms: (a) chelation of calcium ions via surface carboxyl groups, which interferes with the coagulation cascade, and (b) suppression of platelet activation by reducing intracellular calcium levels. The MNPs exhibited uniform spherical morphology with a core size of approximately 14.5 ± 3.5 nm, as confirmed by transmission electron microscopy. They showed selective uptake by thrombin-stimulated platelets, enabling targeted regulation of platelet function. Once internalized, the nanoparticles significantly inhibited thrombin-induced cytoplasmic calcium elevation, a key driver of platelet activation and aggregation. Furthermore, coagulation assays demonstrated that these MNPs effectively prolonged clotting time, indicating inhibition of both intrinsic and extrinsic coagulation pathways. Under oscillating magnetic fields, the MNPs generated mechanical forces capable of disrupting the fibrin network, thereby enhancing thrombolytic activity in both *in vitro* and *in vivo* settings.

Compared to HOOC-PEG2000-MNPs, which rely on external magnetic fields and calcium chelation for targeting and thrombolysis, the low molecular weight heparin-octadecyl amine (LMWH-ODA) nano-anticoagulant offers a self-assembling, carrier-free alternative that functions independently of magnetic manipulation (Lee et al., 2024). This system was constructed by conjugating LMWH with ODA, enabling spontaneous formation of stable nanoparticles (∼105 nm) in aqueous solution.

The amphiphilic nature of LMWH-ODA facilitated efficient albumin binding, mimicking a lipid shuttle mechanism that prolonged systemic circulation. Unlike conventional LMWH with a short half-life, this nanoformulation (LMHO) retained 97% of the anticoagulant activity and remained pharmacologically active for 4–5 days, substantially reducing dosing frequency.

Molecular dynamics simulations and TEM analysis confirmed the structural stability and bioavailability of LMHO, and demonstrated its strong interaction with albumin, which shielded the drug from enzymatic degradation. *In vivo* pharmacokinetic studies showed that LMHO achieved a 45-fold extension in half-life and an 8.4-fold increase in bioavailability compared to unmodified LMWH, with no signs of systemic toxicity. Moreover, the anticoagulant effect remained reversible via protamine neutralization, ensuring safety in clinical application.

It should be noted that the current evidence for LMWH-ODA nanoparticles is not derived from ischemic stroke models. Rather, their relevance to stroke prevention lies in their improved pharmacokinetic stability and prolonged anticoagulant activity, which may be particularly beneficial for patients at high risk of thromboembolic events such as atrial fibrillation. From a translational perspective, such long-acting nano-anticoagulant systems could potentially reduce dosing frequency and improve adherence in long-term stroke prevention strategies. However, further validation in stroke-relevant models is required to determine their effects on embolic prevention, intracranial bleeding risk, and compatibility with current cerebrovascular prevention regimens.

From the perspective of spatiotemporal control, nanotechnology offers clear advantages over conventional preventive pharmacotherapy. Spatially targeted delivery systems can improve drug accumulation in relevant organs such as the liver or vascular endothelium while reducing systemic exposure. Temporally, sustained-release nanocarriers enable stable long-term drug levels, minimizing fluctuations in blood pressure, lipid concentrations, or platelet activity. Such controlled intervention improves both safety and efficacy, illustrating how nanomedicine can regulate chronic stroke risk factors with greater precision than traditional drug administration.

Taken together, emerging nanotechnology-based strategies provide alternative approaches for the modulation of major modifiable risk factors associated with ischemic stroke prevention, including hypertension, dyslipidemia, platelet activation, and coagulation dysfunction. Representative nano-platforms developed for these preventive purposes are summarized in Table 1.

**TABLE 1:** Representative nanoplatforms for risk factor modulation in the prevention stage of ischemic stroke.

Prevention category	Nanoparticle type	Loaded agent	Targeting mechanism	Key advantage	Stage of development
Antihypertensive	Polymeric nanoparticles (PCL, PLGA, Eudragit)	Nifedipine	Diffusion-controlled sustained release via biodegradable polymer matrix	Improves pharmacokinetic stability and minimizes blood pressure variability	Preclinical animal study
Antihypertensive	Pluronic F68/PVA nanocapsules	Nifedipine	Stabilized nanocapsule system enabling controlled release	Enhanced bioavailability and smoother antihypertensive effect	Preclinical animal study
Lipid-lowering	PLGA nanoparticles	Rosuvastatin and ezetimibe	Passive systemic delivery with improved dissolution	Increased oral bioavailability and sustained lipid reduction	Preclinical
Lipid-lowering	Lipid-polymer hybrid nanoparticles (LPHNPs)	Rosuvastatin and ezetimibe	Liver-preferential accumulation via lipid-PEG surface modification	Improved hepatic targeting and reduced systemic exposure	Preclinical
Antiplatelet	Gold nanoparticles (size-tunable)	None (intrinsic nanozyme effect)	Surface-mediated modulation of platelet activation	Size-dependent regulation of coagulation dynamics	*In vitro* and preclinical study
Antiplatelet	Cell-membrane coated nanoparticles (CM-NPs)	Decoy P2Y12 receptors	Biomimetic receptor-mediated sequestration of antiplatelet drugs	Reversal of excessive platelet inhibition with reduced bleeding risk	Preclinical
Anticoagulant	Carboxyl-PEG modified Fe_3_O_4_ magnetic nanoparticles	None (surface-functionalized MNP)	Magnetic guidance and platelet-selective uptake	Targeted anticoagulation with enhanced thrombolytic potential	Preclinical
Anticoagulant	LMWH-ODA self-assembled nanoparticles	LMWH	Albumin-binding-mediated prolonged circulation	Sustained anticoagulant efficacy with extended systemic exposure	Preclinical

Importantly, different nanoplatforms used in the prevention stage present distinct advantages and limitations. Polymeric nanoparticles such as PLGA-based carriers provide good biocompatibility and controlled drug release, making them attractive for long-term preventive therapy. However, their relatively nonspecific biodistribution may limit targeting precision. In contrast, biomimetic or ligand-modified nanoparticles can improve vascular targeting and prolong circulation time, thereby enhancing therapeutic efficacy. Nevertheless, these systems often involve more complex fabrication processes and may face challenges related to large-scale manufacturing and regulatory translation. Therefore, future preventive nanomedicine strategies should aim to balance targeting specificity, biosafety, and translational feasibility.

### 2.2 Neuroprotection stage

Following the prevention stage, the neuroprotection stage serves as a bridge before reperfusion therapies, focusing on reducing brain tissue damage and extending the therapeutic window for subsequent interventions (Wang et al., 2024d). During this stage, strategies aim to protect the ischemic penumbra and delay irreversible neuronal injury by suppressing key pathological processes such as excitotoxicity, oxidative stress, and inflammation (Vidale et al., 2017; Rajkovic et al., 2018; Shi et al., 2019; Link et al., 2020). Conventional treatments like edaravone and nimodipine have been used to mitigate secondary injury but are often limited by poor BBB penetration and non-specific distribution, leading to reduced efficacy and systemic side effects (Fang et al., 2014; Liu et al., 2019). Importantly, these agents primarily modulate individual pathways and are insufficient to comprehensively regulate the multifactorial injury cascade that evolves during the neuroprotection stage.

**FIGURE 5:**
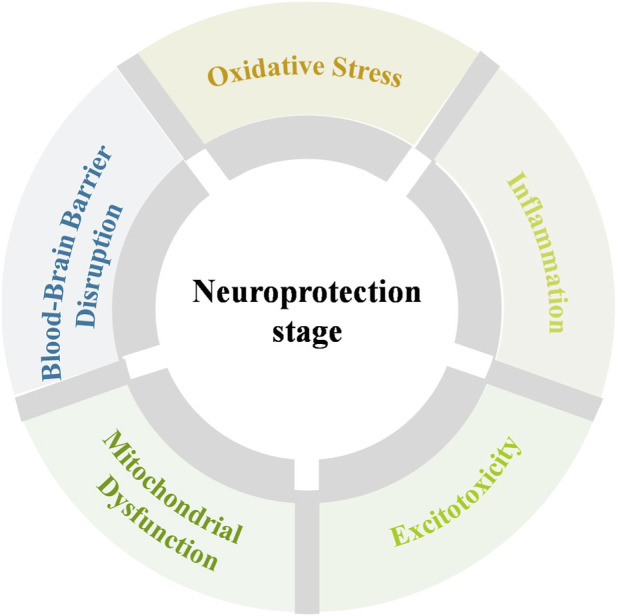
Nanotherapeutics in neuroprotection stage of ischemic stroke.

As summarized in Figure 5, the neuroprotection stage is characterized by a tightly coupled pathological network involving oxidative stress, excitotoxicity, neuroinflammation, mitochondrial dysfunction, and BBB disruption. Importantly, this section focuses on mechanism-driven neuroprotective strategies targeting the ischemic cascade, whereas the regulation of systemic physiological parameters after reperfusion is discussed separately in the adjunctive stage. The dynamic interplay among these processes amplifies neuronal injury and narrows the therapeutic window.

**FIGURE 6:**
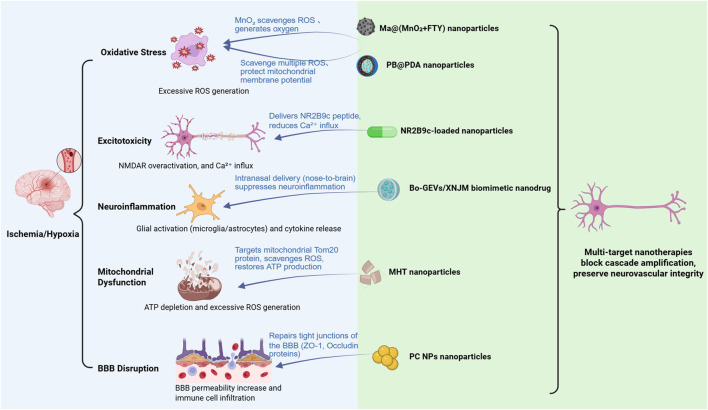
Multi-target nanotherapies for neuroprotection in ischemic stroke. This figure illustrates the multi-target nanotherapies that intervene in key pathological mechanisms during the neuroprotection stage of ischemic stroke. These include the reduction of oxidative stress, suppression of excitotoxicity, modulation of neuroinflammation, restoration of mitochondrial function, and protection of blood-brain barrier integrity. Various nanoparticle-based therapeutic agents are depicted targeting these processes, such as Ma@(MnO_2_+FTY) nanoparticles for oxidative stress, NR2B9c-loaded nanoparticles for excitotoxicity, Bo-GEVs/XNJM biomimetic nanodrug for neuroinflammation, and MHT nanoparticles for mitochondrial protection and BBB repair. These multi-target interventions aim to block cascade amplification and preserve neurovascular integrity.

Building upon this mechanistic framework, nanoparticle-based approaches enable targeted delivery of neuroprotective agents across the BBB and provide precise intervention at specific molecular and subcellular levels. Representative nanotherapeutic strategies targeting these interconnected pathological processes are schematically illustrated in Figure 6.

BBB traversal strategies commonly rely on receptor-mediated transcytosis (RMT) or adsorptive-mediated transcytosis (AMT). RMT-based systems exploit ligand–receptor interactions on brain endothelial cells, enabling relatively high targeting specificity and controlled delivery of therapeutic agents. However, receptor availability and potential receptor saturation may limit the overall transport efficiency of this pathway, particularly when high nanoparticle doses are required. In contrast, AMT relies on electrostatic interactions between positively charged nanocarriers and negatively charged endothelial membranes, allowing more efficient BBB penetration but often at the expense of cellular specificity and increased nonspecific uptake. Overall, receptor-mediated transport offers higher targeting specificity but may be limited by receptor availability and potential saturation, whereas adsorptive-mediated transport generally achieves greater BBB penetration efficiency but with reduced cellular selectivity. Despite the extensive development of neuroprotective nanoplatforms, a critical challenge lies in the temporal mismatch between the rapid progression of ischemic injury and the pharmacokinetic onset of many nanoparticle systems. Therefore, the selection of BBB-crossing strategies should balance targeting precision and transport efficiency, depending on the therapeutic objective and the pathological stage of stroke intervention.

#### 2.2.1 Reducing oxidative stress

**FIGURE 7:**
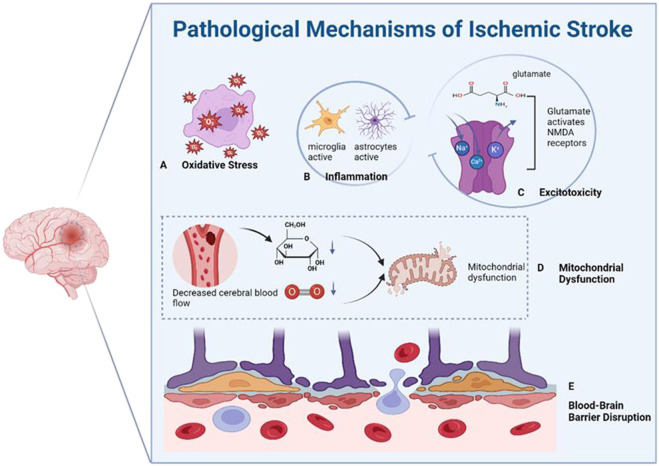
Primary pathological mechanisms of ischemic stroke. **(A)** Oxidative stress. **(B)** Inflammation. **(C)** Excitotoxicity. **(D)** Mitochondrial dysfunction. **(E)** Disruption of the blood-brain barrier (BBB).

Oxidative stress plays a significant role in ischemic stroke by directly inducing neuronal injury and amplifying other pathological processes (Figure 7A) (Suh et al., 2008; Choi et al., 2009; Allen and Bayraktutan, 2009). Excessive generation of reactive oxygen species (ROS), such as superoxide anion, hydroxyl radical, and nitric oxide, leads to damage of Deoxyribonucleic Acid (DNA), proteins, and lipids (Love, 1999; Neumar, 2000; Yamamoto et al., 2008; Ridder and Schwaninger, 2009; Chong et al., 2005; Luo and Sun, 2007; Weinstein et al., 2004; Kiewert et al., 2010; Sumbria et al., 2011).

Traditional antioxidant therapies have shown limited efficacy due to poor bioavailability and insufficient mitochondrial targeting (Martinelli et al., 2020). To combat oxidative stress in ischemic stroke, various nanoparticle-based antioxidant strategies have been developed to improve the targeting and efficacy of conventional treatments. Li et al. constructed macrophage membrane-coated manganese dioxide nanoparticles loaded with fingolimod [Ma@(MnO_2_+FTY)] (Li et al., 2021a). These nanoparticles exerted dual effects: the MnO_2_ core reacted with hydrogen peroxide to eliminate ROS and generate oxygen, relieving local oxidative stress and hypoxia; meanwhile, the macrophage membrane enabled accumulation in ischemic brain regions through interactions with adhesion molecules on damaged endothelial cells. Fingolimod further modulated the inflammatory microenvironment by promoting microglial polarization toward the M2 phenotype and inhibiting nuclear factor-kappa B (NF-κB) signaling. However, although Ma@(MnO_2_+FTY) demonstrated biomimetic targeting at the tissue level, it lacked subcellular specificity and could not effectively eliminate ROS at their primary source within mitochondria.

In contrast, Zhao et al. developed Prussian blue@polydopamine (PB@PDA) nanoparticles with enhanced mitochondrial targeting (Zhao et al., 2024). The Prussian blue core provided catalase-, superoxide dismutase-, and peroxidase-like activities to neutralize multiple ROS species, while the polydopamine coating stabilized the nanoparticles and facilitated selective accumulation within neuronal mitochondria. By directly scavenging mitochondrial ROS and preserving membrane potential, PB@PDA nanoparticles more effectively prevented oxidative damage and neuronal apoptosis compared to untargeted counterparts.

However, excessive suppression of reactive oxygen species may interfere with physiological redox signaling that contributes to endogenous repair mechanisms, indicating that therapeutic modulation of oxidative stress should aim for balance rather than maximal elimination.

#### 2.2.2 Anti-inflammatory therapy

Ischemic stroke initiates a robust inflammatory response, which plays a key pathological role in both acute injury and long-term neurological deficits (Shi et al., 2019). Damage-associated molecular patterns (DAMPs) released from necrotic cells can activate resident immune cells, including particularly microglia and astrocytes, further triggering the production of pro-inflammatory cytokines and chemokines (Figure 7B) (Gadani et al., 2015; Liesz et al., 2015; Shichita et al., 2017; Li et al., 2017; Burda et al., 2016; Hayakawa et al., 2016). This localized neuroinflammation further exacerbates oxidative stress and excitotoxicity, while systemic propagation of inflammation contributes to BBB disruption and secondary neuronal damage. Thus, mitigating post-stroke inflammation is critical for preserving neurological function and improving recovery.

Current anti-inflammatory therapies include corticosteroids, non-steroidal anti-inflammatory drugs (NSAIDs), and biologics targeting inflammatory pathways. However, their effectiveness is limited by poor BBB permeability, off-target effects, and short half-life (Cui et al., 2025). These limitations have driven increasing interest in nanomaterial-based strategies that enhance brain delivery efficiency, enable localized accumulation, and provide sustained anti-inflammatory effects. Two recent studies by Yuan et al. and Li et al. highlight innovative nano-interventions to address these challenges.

Yuan et al. designed TPCD nanoparticles by conjugating β-cyclodextrin with Tempol and phenylboronic acid pinacol ester (PBAP), enabling dual scavenging of superoxide and hydrogen peroxide (Yuan et al., 2021). This multifunctional design allows simultaneous modulation of oxidative stress and inflammation in ischemic brain tissue. In experimental middle cerebral artery occlusion (MCAO) models, systemically administered TPCD nanoparticles exhibited preferential accumulation in ischemic regions and effectively suppressed intracellular ROS levels in activated microglia. Consistent with these cellular effects, TPCD treatment significantly reduced infarct volume and improved neuroprotection *in vivo*. However, the therapeutic efficacy of this platform remains constrained by passive BBB penetration, relatively rapid systemic clearance, and the requirement for repeated intravenous administration.

To address these shortcomings, Li et al. developed a nasal-administered biomimetic nanodrug (Bo-GEVs/XNJM) that bypasses the BBB and enables direct nose-to-brain delivery of anti-inflammatory agents (Li et al., 2024a). Unlike TPCD nanoparticles, which depend on systemic circulation for brain access, this strategy exploits the olfactory and trigeminal pathways to achieve more efficient and targeted central nervous system delivery. The system integrates grapefruit-derived extracellular vesicles (GEVs) with Xingnaojing microemulsion (XNJM) and is further modified with borneol to enhance mucosal penetration and transport efficiency. Physicochemical characterization confirmed the structural stability of the formulation, while in MCAO models, intranasal administration significantly reduced infarct volume and suppressed pro-inflammatory cytokine release and oxidative stress markers. This approach provides a non-invasive alternative with improved brain bioavailability, reduced systemic exposure, and enhanced translational potential for post-stroke anti-inflammatory therapy.

#### 2.2.3 Targeting excitotoxicity

Excitotoxicity is a major contributor to neuronal death following ischemic stroke, driven by impaired ionic homeostasis and excessive activation of N-methyl-D-aspartate receptors (NMDARs) (Figure 7C) (van Putten et al., 2021; Harris et al., 2012; Krzyżanowska et al., 2014; Sattler et al., 1999) Energy depletion disrupts ion pumps, causing abnormal glutamate accumulation in the synaptic cleft, which overactivates NMDARs and triggers massive calcium influx. This initiates a cascade of calcium-dependent enzyme activation, mitochondrial dysfunction, and ultimately leads to necrosis, apoptosis, and autophagy (Kornau et al., 1995; Tochio et al., 2000; Du et al., 2009; Hou et al., 2005; Hou et al., 2002; Wang et al., 2010; Shen et al., 2022). While pharmacological interventions such as NMDAR antagonists and calcium channel blockers aim to alleviate excitotoxicity, their clinical use is limited by poor BBB permeability and non-specific inhibition of synaptic NMDARs, which are essential for normal neuronal signaling and plasticity (Kandy et al., 2022).

To address these challenges, Li et al. developed a nasal-delivered nanoparticle system to deliver the neuroprotective peptide NR2B9c and selectively disrupt pathological NMDAR signaling (Li R. et al., 2019). NR2B9c inhibits the interaction between NMDARs and postsynaptic density protein-95 (PSD-95), thereby blocking downstream excitotoxic cascades without affecting normal synaptic transmission. The resulting nanoparticles exhibited good colloidal stability and neuronal affinity through surface modification with wheat germ agglutinin (WGA), which enhanced mucosal transport and neuronal uptake. Cellular uptake studies demonstrated that WGA functionalization significantly improved NR2B9c delivery to neurons and epithelial cells, supporting efficient nose-to-brain transport. *In vivo*, NR2B9c-loaded nanoparticles markedly reduced infarct volume and promoted functional recovery in experimental stroke models. However, this strategy may still affect both synaptic and extrasynaptic NMDAR populations, raising concerns about potential interference with physiological neurotransmission.

To achieve more precise receptor targeting, Valente et al. developed gold nanoparticles (AuNPs) functionalized with subtype-selective conopeptides (Con-G and Con-R) that antagonize extrasynaptic NMDARs containing GluN2B subunits while sparing synaptic receptors (Valente et al., 2020). By engineering the nanoparticles to exceed the size limit of the synaptic cleft, their activity was spatially restricted to extrasynaptic receptors, minimizing disruption of normal synaptic signaling. Electrophysiological analyses confirmed that conopeptide-AuNPs selectively suppressed extrasynaptic NMDAR-mediated currents without altering synaptic transmission. *In vitro* neurotoxicity assays further demonstrated that these nanoparticles significantly attenuated NMDA-induced neuronal death, highlighting their selective neuroprotective efficacy. This receptor- and localization-specific strategy offers a refined nanotherapeutic approach to mitigating excitotoxic injury while preserving essential neuronal communication.

#### 2.2.4 Mitochondrial protection

Mitochondrial dysfunction is a key pathological mechanism in ischemic stroke. Oxygen and glucose deprivation impairs oxidative phosphorylation, resulting in ATP depletion, excessive ROS generation, and oxidative injury to cellular components (Figure 7D) (An et al., 2021; Anderson and Sims, 1999; Chouchani et al., 2014; Li et al., 2010; Ferrer et al., 2003; Bulthuis et al., 2019; Kondadi et al., 2019; Murley and Nunnari, 2016) Damaged mitochondria also activate apoptosis pathways, exacerbating neuronal loss (Chan, 2004). Therefore, restoring mitochondrial function is crucial for effective neuroprotection. Traditional treatments, such as antioxidants and metabolic support, are limited by poor BBB penetration and inefficient mitochondrial targeting (Novorolsky et al., 2023).

To address this, Li et al. developed cerium oxide nanoparticles loaded with dl-3-n-butylphthalide (NBP-CeO_2_ NPs), which integrate catalytic ROS scavenging with neurovascular protection (Li et al., 2022a). These ultrasmall (∼5 nm), PEGylated nanoparticles were synthesized via high-temperature decomposition, and their nanoscale size and uniform morphology enabled efficient redox activity and favorable biological distribution. Mechanistically, CeO_2_ nanoparticles act as regenerative antioxidants by cycling between Ce^3+^ and Ce^4+^ states, continuously eliminating ROS such as superoxide and hydroxyl radicals. Meanwhile, dl-3-n-butylphthalide (NBP) contributes to mitochondrial stabilization and promotes angiogenesis, further supporting neurovascular recovery.

*In vitro* studies in brain microvascular endothelial cells (BMVECs) demonstrated that NBP-CeO_2_ NPs effectively reduced intracellular ROS levels and preserved mitochondrial membrane potential, indicating protection against oxidative mitochondrial damage. *In vivo*, systemic administration of these nanoparticles significantly reduced infarct volume, improved neurological outcomes, and preserved BBB integrity in middle cerebral artery occlusion/reperfusion (MCAO/R) models. However, their therapeutic efficacy was constrained by limited subcellular specificity, as mitochondrial accumulation relied primarily on passive intracellular distribution rather than active mitochondrial targeting.

To improve mitochondrial specificity, Wang et al. developed Melanin-Heteropolyacid-Tannic (MHT) acid nanoparticles composed of melanin-modified heteropolyacid and tannic acid (TA), which were designed for sequential tissue-to-organelle targeting (Wang et al., 2024c). TA facilitates BBB penetration by binding to ischemia-exposed extracellular matrix proteins, whereas mitochondrial accumulation is achieved through interaction with Tom20 proteins on the outer mitochondrial membrane. Although this system was evaluated in a cerebral ischemia–reperfusion injury (CIRI) model, the pathological mechanisms targeted by this strategy, including mitochondrial ROS accumulation and inflammation-associated signaling, are already activated during the ischemic stage, rendering it directly relevant to early neuroprotection. Importantly, this design overcomes the subcellular targeting limitations observed in the NBP-CeO_2_ system by enabling direct and selective delivery to neuronal mitochondria. Unlike CeO_2_-based systems, MHT nanoparticles provide mitochondrial protection by simultaneously scavenging ROS, restoring ATP production, and suppressing activation of the cGAS–STING pathway triggered by mitochondrial DNA release. In CIRI models, MHT treatment markedly reduced infarct size and preserved brain structure at a low dose of 2 mg/kg, demonstrating superior efficacy compared with conventional antioxidants.

#### 2.2.5 Blood-brain barrier protection

BBB dysfunction plays a central role in ischemic stroke pathology. In the initial stages of stroke, the intact BBB severely limits the entry of therapeutic agents into the brain, delaying timely neuroprotection. As ischemia progresses, the BBB becomes increasingly permeable due to tight junction degradation and endothelial damage, allowing excessive infiltration of immune cells and pro-oxidant molecules. This breakdown contributes to neuroinflammation, edema, and secondary neuronal injury (Figure 7E) (Li et al., 2021b; Huang et al., 2023; Kim et al., 2025) Persistent BBB disruption further impairs brain homeostasis and increases the risk of long-term neurological decline (Szarka et al., 2019).

Traditional treatment strategies, including anti-inflammatory and antioxidant agents, aim to protect the BBB by suppressing inflammation and oxidative damage. However, these therapies often show limited efficacy due to poor BBB penetration, rapid systemic clearance, and a lack of direct endothelial repair mechanisms. These limitations highlight the need for advanced therapeutic platforms capable of both penetrating the BBB and actively restoring its structural integrity.

To address this, Gao et al. developed mannose-functionalized lipid nanoparticles (MLNPs) for targeted delivery of IL-10 mRNA to M2-polarized microglia via the CD206 receptor (Gao et al., 2024). This strategy enhanced IL-10 expression, promoted anti-inflammatory microglial polarization, and reduced the production of TNF-α, IL-6, and iNOS. Particle characterization confirmed a uniform size distribution and stable colloidal properties. *In vivo* studies showed reduced IgG extravasation, indicating alleviation of BBB leakage, together with improved neuronal integrity, decreased infarct volume, and improved neurological outcomes. Although this approach alleviated BBB leakage, its effect was mediated through immune modulation rather than direct structural repair of the BBB.

To achieve more direct endothelial restoration, Liu et al. designed cerium-doped myricetin oligomer nanoparticles (PC NPs), which utilize multi-receptor-mediated transcytosis to enhance BBB penetration and directly target endothelial cells (Liu et al., 2024a). These nanoparticles activated protective autophagy and promoted tight junction protein restoration. Western blot analysis showed increased expression of ZO-1, Occludin, and Claudin-5 compared to the untreated group, indicating enhanced endothelial barrier repair. Compared with free myricetin and non-doped controls, PC NPs achieved stronger neuroprotection in early stroke models by combining BBB penetration with structural restoration.

Beyond the examples discussed above, a variety of nanostrategies have been developed to improve drug delivery across the BBB, including sustained-release nanocarriers, lipid-based delivery systems, stimuli-responsive nanostructures, surface-engineered nanocarriers, and intranasal nanodelivery approaches. Representative strategies and their mechanisms are summarized in Table 2 (Cunha et al., 2021; Rafati et al., 2024; Manimaran et al., 2023; McLoughlin et al., 2024; Adscheid et al., 2024).

**TABLE 2:** Nanoparticle-based approaches for BBB protection, repair, and delivery in ischemic stroke.

Strategy	Mechanism of action	Outcome/Advantage
Polymeric nanoparticles (PNPs)	Sustained and localized delivery of therapeutic agents targeting BBB dysfunction	Attenuates BBB permeability changes and supports BBB integrity
Lipid nanoparticles (LNPs)	Lipid matrix enables BBB penetration while protecting mRNA or other fragile therapeutics	Facilitates efficient delivery of therapeutic cargo across the BBB
Nanogels and micelles	Adaptive nanostructures facilitating enhanced BBB permeability and controlled drug release	Prolonged circulation and enhanced brain accumulation
Carbon-based nanoparticles (graphene, carbon nanotubes)	Surface functionalization enables receptor-mediated transcytosis or passive diffusion across the BBB	Enhances brain-targeted delivery with potential neuroprotective effects
Nasal delivery nanoparticles	Bypasses BBB via olfactory and trigeminal nerve pathways, ensuring rapid drug delivery to the brain	Reduces systemic distribution and ensures effective brain drug delivery

Importantly, the permeability of the blood-brain barrier evolves dynamically during stroke progression. Nanocarrier designs optimized for an intact barrier may therefore differ substantially from those required when the barrier becomes disrupted. This dynamic change highlights the importance of stage-adaptive delivery strategies in neuroprotective nanomedicine.

Collectively, these nanotechnology-based neuroprotective strategies demonstrate how spatiotemporal control can be applied to intervene in the complex cascade of ischemic injury. Spatial targeting enables nanocarriers to cross the BBB and accumulate in specific cellular compartments such as neurons, microglia, or mitochondria. Temporally controlled release further allows therapeutic agents to be delivered during the early stages of ischemic injury when neuroprotective intervention is most effective. This combination of spatial precision and temporal synchronization represents a major advantage of nanomedicine compared with conventional neuroprotective drugs.

To facilitate stage-level comparison across these mechanisms, representative nanocarriers developed for the neuroprotection stage are summarized in Table 3.

**TABLE 3:** Representative nanocarriers for neuroprotection in the neuroprotection stage of ischemic stroke.

Nanocarrier	Active cargo	Targeting strategy	Primary mechanism of action	Key experimental findings
Macrophage membrane-coated MnO_2_ nanoparticles	Fingolimod	Biomimetic inflammation-site targeting	Reactive oxygen species scavenging and immune modulation	Reduced infarct size and promoted anti-inflammatory microglial polarization
PB@PDA nanoparticles	Enzyme-mimetic core	Mitochondria-associated accumulation	Multi-pathway antioxidant activity and mitochondrial protection	Preserved mitochondrial function and attenuated neuronal injury
TPCD nanoparticles	Tempol-cyclodextrin	Passive ischemic-region accumulation	Combined antioxidant and anti-inflammatory effects	Reduced oxidative stress and infarct volume
WGA-modified nanoparticles	NR2B9c peptide	Neuronal targeting via lectin-mediated uptake	Disruption of excitotoxic NMDAR-PSD95 signaling	Reduced excitotoxic neuronal damage and infarct size
NBP-CeO_2_ nanoparticles	dl-3-n-butylphthalide	Reactive oxygen species-responsive delivery	Antioxidant activity and mitochondrial functional preservation	Improved neurological recovery in experimental stroke
MHT nanoparticles	Tannic-acid-modified heteropolyacid	Blood-brain barrier penetration and mitochondrial targeting	Reactive oxygen species scavenging and ATP restoration	Reduced infarct volume and improved energy metabolism
Mannose-modified lipid nanoparticles	IL-10 mRNA	CD206-positive microglia targeting	Anti-inflammatory polarization and vascular protection	Reduced neuroinflammation and blood-brain barrier leakage

In addition to BBB transport strategies, different neuroprotective nanoplatforms exhibit distinct functional advantages depending on the targeted pathological process. Enzyme-mimicking nanoparticles such as Prussian blue or cerium oxide mainly exert therapeutic effects through catalytic scavenging of reactive oxygen species, providing sustained antioxidant activity but sometimes lacking precise cellular targeting. In contrast, receptor-targeted or biomimetic nanocarriers can achieve improved cell-type specificity and enhanced accumulation in ischemic regions, although their therapeutic efficiency may depend on receptor expression levels and pathological conditions. Consequently, integrating catalytic nanomaterials with targeted delivery strategies may represent a promising approach for achieving both efficient ROS elimination and precise neuroprotection.

### 2.3 Revascularization stage

Following the prevention and neuroprotection stages, revascularization becomes the immediate therapeutic priority in ischemic stroke, aiming to promptly restore CBF, limit neuronal damage, and improve clinical outcomes (Wassélius et al., 2022). If reperfusion is delayed, sustained ischemia will trigger a cascade of injury mechanisms—including ATP depletion, ionic imbalance, excitotoxicity, oxidative stress, inflammation, and BBB breakdown, which will further expand the infarct core and worsen neurological prognosis (Schoknecht et al., 2024; Abadin et al., 2024; Salsano et al., 2024).

Among current strategies, intravenous thrombolysis remains widely used, but its application is limited by a narrow time window and risks such as hemorrhagic transformation and treatment delay (Jia et al., 2021). To address these issues from a clinical perspective, our team has focused on optimizing patient selection to minimize complications. Our research suggests that, for appropriately screened patients, direct MT without prior thrombolysis can provide comparable or even improved outcomes, particularly in achieving functional independence within 90 days (Zi et al., 2021). This approach helps reduce bleeding risk and treatment delays.

Nonetheless, many patients continue to experience suboptimal recovery, highlighting the limitations of current reperfusion therapies. In this context, integrating nanomaterials with thrombolytic and endovascular interventions offers promising solutions. By enabling precise thrombus targeting, controlled drug release, and reduced systemic toxicity, nanotechnology holds potential to enhance the safety and efficacy of revascularization treatments in ischemic stroke.

#### 2.3.1 Intravenous thrombolysis

Intravenous thrombolysis remains a cornerstone in acute ischemic stroke treatment, primarily relying on pharmacologic agents such as alteplase, reteplase, and tenecteplase to dissolve clots and restore cerebral blood flow (Tsivgoulis et al., 2017; Ye et al., 2022; Otite et al., 2021; Aguiar de Sousa et al., 2019; Bach and Lui, 2024). Despite their clinical utility, these agents face notable limitations: they must be administered within a narrow time window (typically within 4.5 h of symptom onset), are delivered systemically—thereby lacking thrombus specificity and increasing the risk of hemorrhagic complications—and exhibit poor retention at the clot site, limiting local efficacy (Shen et al., 2023). These drawbacks significantly hinder the overall safety and effectiveness of thrombolytic therapy.

To address these issues, researchers have explored nanoparticle-based delivery systems that enable targeted thrombolysis with enhanced precision, reduced systemic toxicity, and controlled drug release.

Li et al. developed a magnetically responsive thrombolytic system by covalently conjugating urokinase to Fe_3_O_4_ nanoparticles (Li Q. et al., 2019). The nanoparticles were functionalized with oleic acid (OA) and poly (maleic anhydride alt 1 octadecene) (PMAO) to improve dispersibility and stability. Following administration, the nanoparticles accumulated at the thrombus site under a static magnetic field, leading to increased local drug concentration and reduced off target exposure. Subsequent application of an alternating magnetic field triggered accelerated urokinase release, resulting in a marked enhancement of thrombolytic efficiency compared with passive release conditions. However, the therapeutic performance of this system remains dependent on external magnetic field application, which may pose practical challenges for clinical translation.

To overcome these limitations, De La Taille et al. designed Fucoidan modified polysaccharide nanoparticles (Fuco NPs) that achieve biological thrombus targeting without reliance on external physical guidance (de La Taille et al., 2025). These nanoparticles were synthesized via a microemulsion-based method involving emulsification, crosslinking, and purification to generate uniform, water-dispersible Fuco NPs. Targeting was mediated through specific interaction with P-selectin expressed at the thrombus site, enabling precise localization under physiological conditions. Once localized, Fuco NPs achieved dual-action thrombolysis by co delivering recombinant tissue plasminogen activator (rt PA) and DNase I to synergistically degrade fibrin and neutrophil extracellular traps (NETs), thereby enhancing clot resolution while minimizing fragmentation. *In vivo*, Fuco NPs demonstrated effective thrombolysis at significantly reduced rt PA doses, minimizing hemorrhagic risk and enhancing treatment safety.

In addition to these two representative studies, a variety of nanoparticle-based systems—such as magnetic, lipid, polymeric, and stimuli-responsive platforms—are being actively explored to improve thrombolysis by enhancing clot targeting, prolonging drug retention, and minimizing bleeding risk. These approaches are summarized in Table 4, which outlines representative targeted nanodelivery strategies, their design mechanisms, and therapeutic applications in thrombolytic therapy (Verma, 2024; Gu et al., 2024; Marecki et al., 2024; Liu et al., 2024b; Hu et al., 2022).

**TABLE 4:** Representative nanoparticle strategies for enhancing intravenous thrombolysis in ischemic stroke.

Nanoparticle type	Material composition	Advantages	Applications in thrombolytic therapy
Magnetic NPs	Iron oxide core with functionalized surface	Precise targeting via magnetic fields; reduced systemic side effects	Direct thrombus targeting and enhanced thrombolysis
Lipid NPs	Lipid-based structures	High drug loading capacity; sustained release	Nanocarrier-mediated delivery of thrombolytic agent
Polymeric NPs	Biodegradable polymers like PLGA, PEG	Controlled drug release; biocompatibility	Controlled thrombolytic drug delivery and improved local retention
Stimuli-responsive NPs	Environment-sensitive polymers or lipids	Triggered drug release under specific conditions (e.g., pH, temperature)	Stimulus-triggered drug release at thrombus site
Ultrasound-triggered NPs	Lipid or polymeric nanoparticles with sensitive shells	Precise spatiotemporal control of thrombolysis	Ultrasound-mediated spatiotemporal activation of thrombolytic agents

#### 2.3.2 Endovascular thrombectomy

Endovascular thrombectomy (EVT) has revolutionized the treatment of ischemic stroke by mechanically restoring vascular patency, especially in large vessel occlusions. Techniques such as stent retrievers, aspiration, and intra-arterial thrombolysis have significantly improved patient outcomes, allowing for extended treatment windows and more personalized interventions based on advanced imaging (Rabinstein, 2020). However, EVT still faces critical limitations: incomplete recanalization in small or distal vessels, risk of vascular injury from mechanical devices, and challenges in real-time procedural control (Fatehpur et al., 2025; Pilgram-Pastor et al., 2021). Moreover, access to EVT is limited in many regions, potentially delaying treatment.

Nanotechnology offers promising solutions to enhance EVT precision, efficacy, and safety. Grayston et al. developed multifunctional nanocarriers (NCs) composed of poly (lactic-co-glycolic acid) (PLGA) encapsulating superparamagnetic iron oxide nanoparticles (SPIONs) and Cy7.5 dye, enabling magnetic guidance and multimodal imaging (Grayston et al., 2022). These nanocarriers were administered via intra-arterial infusion and guided to the ischemic hemisphere using a static magnetic field. *In vivo* and *ex vivo* fluorescence imaging demonstrated significantly higher brain accumulation with magnetic targeting compared to intravenous or non-guided approaches. Moreover, T2-weighted MRI at 48 h confirmed enhanced nanoparticle retention in the ipsilateral hemisphere under magnetic guidance, supporting the platform’s stability and therapeutic potential. Importantly, no increase in infarct volume or hemorrhage was observed, indicating good biocompatibility. While this study achieved targeted delivery, it did not address real-time procedural monitoring.

To bridge this gap, Jyoti et al. introduced a real-time thrombus monitoring system based on antibody-functionalized magnetic nanoparticles that selectively bind to thrombi (Jyoti et al., 2025). These nanoparticles generate detectable magnetic flux changes that can be sensed by a fine-gauge pickup coil attached to the stent retriever. Simulation and experimental validation demonstrated high spatial precision in thrombus localization, achieving micrometer-scale resolution at short detection distances. This sensing mechanism allows real-time tracking of clot position and slippage during mechanical retrieval, representing a major advance over conventional imaging approaches that lack procedural feedback. By enabling dynamic adjustment of retrieval force and technique, this system minimizes clot fragmentation and embolization risk.

**FIGURE 8:**
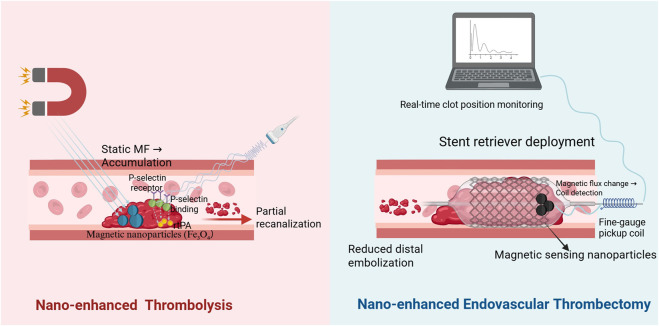
Smart revascularization strategies integrating nano-enhanced thrombolysis and nano-enhanced endovascular thrombectomy in ischemic stroke. (Left) Nano-enhanced thrombolysis. Magnetic nanoparticles (Fe_3_O_4_) accumulate at the thrombus site under a static magnetic field, enabling spatial targeting and increased local drug concentration. Fucoidan-modified nanoparticles further enhance thrombus specificity through P-selectin–mediated binding. Upon external stimulation (e.g., ultrasound), thrombolytic agents such as rtPA are locally released, promoting fibrin degradation and partial recanalization while minimizing systemic exposure. (Right) Nano-enhanced endovascular thrombectomy. Magnetic sensing nanoparticles selectively bind to thrombi and generate detectable magnetic flux changes during mechanical retrieval. A fine-gauge pickup coil integrated with the stent retriever enables real-time clot position monitoring, allowing dynamic procedural adjustment and reducing distal embolization risk. Together, these approaches illustrate how nanotechnology enables spatial targeting, temporally controlled intervention, and real-time feedback during the revascularization stage of ischemic stroke.

Beyond these two representative studies, researchers have developed diverse nanoparticle strategies to address various limitations of mechanical thrombectomy. These efforts aim to improve clot localization, enhance retrieval precision, and minimize complications such as vascular injury or distal embolization. As summarized in Table 5, a range of nano-enabled strategies have been developed to improve clot targeting, disruption, and retrieval during thrombectomy, including magnetically guided nanoparticle swarms, photothermal nanomaterials, biomimetic drug-delivery systems, and antithrombotic nanotube coatings (Manamanchaiyaporn et al., 2021; Dong et al., 2018; Liu et al., 2023; Zhang et al., 2020). These platforms enhance clot targeting, improve retrieval efficiency, and reduce procedural complications, collectively advancing the precision and safety of endovascular interventions. In the revascularization stage, the concept of spatiotemporal control is particularly evident. Spatial targeting enables nanocarriers to localize directly at thrombi or occluded vessels, significantly enhancing the efficiency of thrombolysis or thrombectomy. Meanwhile, temporally responsive systems can release thrombolytic agents only upon external stimulation or in response to the thrombus microenvironment, thereby minimizing systemic bleeding risks. These capabilities illustrate how nanomedicine can enhance the precision and controllability of conventional reperfusion strategies. To provide a conceptual overview of these integrated strategies, the mechanisms of nano-enhanced thrombolysis and nano-enhanced endovascular thrombectomy during the revascularization stage are illustrated in Figure 8. Currently, most nanoparticle-based thrombolytic systems are explored as adjunctive approaches to improve pharmacological thrombolysis rather than to replace mechanical thrombectomy. While these systems improve drug targeting and retention at thrombus sites, their clinical superiority over optimized mechanical thrombectomy remains to be demonstrated in large-scale comparative studies.

**TABLE 5:** Comparison of nanoparticle strategies for enhancing mechanical thrombectomy.

Nanoparticle type	Material composition	Advantages	Applications in thrombectomy
Magnetic nanoparticles	Iron oxide (Fe_3_O_4_), coated with biocompatible polymers	Magnetically guided targeting; high adsorption efficiency	Capturing and removing clots via external magnetic fields
Gold nanorods	Gold nanoparticles, surface functionalized	Photothermal effect for clot softening; biocompatibility	Assisting mechanical thrombectomy by softening clots
Biomimetic nanoparticles	Mimicking platelet structures or vesicles	Targeted adhesion to thrombus; biocompatibility	Enhancing clot capture and stability during removal
Titanium dioxide nanorods (TiO_2_)	Crystalline TiO_2_ with high mechanical stability	Strong physical adsorption; effective barrier against clot fragments	Blocking and collecting clot fragments; supporting thrombectomy devices

Compared with conventional thrombolytic therapy, nanoparticle-based thrombolytic systems offer improved thrombus targeting and reduced systemic bleeding risk. Magnetic nanoparticle systems enable precise spatial control under external magnetic fields, which can significantly enhance local thrombolysis efficiency. However, their clinical application may depend on specialized equipment and external field control. In contrast, biologically targeted nanoparticles, such as ligand-modified or biomimetic nanocarriers, achieve thrombus localization through endogenous molecular recognition, improving clinical feasibility but sometimes with lower spatial controllability. Future thrombolytic nanoplatforms may integrate both biological targeting and physical guidance to maximize recanalization efficiency while maintaining clinical practicality.

### 2.4 Adjunctive stage

**FIGURE 9:**
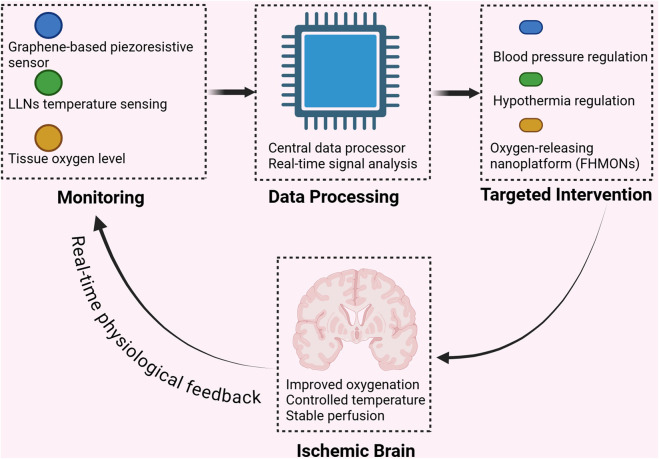
Nanotechnology-enabled closed-loop system for adjunctive management of ischemic stroke. Conceptual illustration of a nanotechnology-enabled closed-loop system for adjunctive management of ischemic stroke. Nanomaterial-based sensing platforms enable real-time monitoring of key physiological parameters, including blood pressure and brain temperature. The collected signals are processed through real-time data analysis to guide targeted interventions such as blood pressure regulation, therapeutic hypothermia, and oxygen delivery using oxygen-releasing nanoplatforms (FHMONs). Continuous physiological feedback allows dynamic adjustment of adjunctive therapies to stabilize cerebral perfusion, control brain temperature, and improve tissue oxygenation following reperfusion.

The adjunctive stage is a critical but often underrecognized component of ischemic stroke management. It follows revascularization and focuses on stabilizing systemic and cerebral homeostasis to support neurological recovery. Despite successful recanalization, many patients still experience poor outcomes due to secondary injuries such as hypoxia, inflammation, and hemorrhagic transformation. Among adjunctive strategies, blood pressure control, therapeutic hypothermia, and oxygen therapy are particularly important, as they target the core pathophysiological processes underlying post-reperfusion injury (Mizuma et al., 2018; Eskla et al., 2022; Sharma, 2023). These interventions aim to protect the brain by reducing oxidative stress, preserving vascular integrity, and improving oxygen delivery. However, conventional methods often lack precision and timely responsiveness. Nanotechnology offers promising solutions by enabling real-time monitoring, targeted delivery, and enhanced therapeutic precision and responsiveness, making it a valuable tool for improving adjunctive care in ischemic stroke (Figure 9). This stage is distinct from neuroprotection in that it focuses on the regulation of physiological parameters post-reperfusion, rather than modulating the ischemic injury cascade.

#### 2.4.1 Blood pressure management

While long-term blood pressure control in the prevention stage aims to reduce the risk of stroke onset, blood pressure regulation in the adjunctive stage emphasizes short-term precision to stabilize cerebral perfusion and reduce the risks of reperfusion-induced injury and intracerebral hemorrhage (ICH), particularly after successful endovascular recanalization (Fanning et al., 2022).

Currently, pharmacological interventions are the primary method for blood pressure control in this stage, aiming to rapidly mitigate hypertension-related risks (Pai et al., 2017). However, achieving optimal blood pressure solely through drug administration remains challenging. These agents often lack the precision needed for real-time regulation, which can result in overcorrection or delayed response, potentially exacerbating neurological injury (Shiffermiller and Whinney, 2023).

This limitation is particularly evident in adjunctive stage following MT, where precise hemodynamic control is essential. Our team’s Second Enhanced Control of Hypertension and Thrombectomy (ENCHANTED-2/MT) trial demonstrated that although intensive antihypertensive therapy (targeting systolic blood pressure <120 mmHg) was intended to reduce reperfusion injury, it was paradoxically associated with poorer functional recovery and increased neurological deterioration (Mazighi et al., 2021; Yang et al., 2022). Based on these findings, a more conservative strategy targeting a systolic range of 140–180 mmHg is now advocated to balance perfusion preservation and bleeding risk, optimizing patient outcomes in the adjunctive stage.

Moreover, conventional cuff-based monitoring lacks the sensitivity to detect rapid blood pressure fluctuations in this dynamic period. To address these shortcomings, nanomaterial-based technologies have emerged as promising alternatives for real-time, continuous, and non-invasive monitoring. To address these shortcomings, nanomaterial-based technologies have emerged as promising alternatives for real-time, continuous, and non-invasive monitoring. For example, flexible graphene-based piezoresistive sensors developed by Zhang et al. demonstrated high-performance detection of arterial pulse changes. These systems utilize a compressible melamine sponge coated with graphene nanomaterials, and as the sponge compresses and relaxes, resistance within the conductive network varies, converting mechanical pressure into electrical signals for accurate blood pressure estimation (Zhang et al., 2022). Compared with pharmacologic or cuff-based approaches, this platform offers superior responsiveness, enabling personalized and fine-tuned blood pressure monitoring in real time. The study demonstrated continuous, rapid-response detection with high sensitivity, showing potential for integration into wearable devices for real-time hemodynamic tracking.

Although Zhang et al. (2022) primarily focused on monitoring rather than active therapeutic regulation, the pressure-sensitive outputs from such nanosensors provide a foundation for potential closed-loop systems. For instance, in future designs, real-time signals from the sensor could be integrated with smart drug delivery platforms, where antihypertensive agents are released adaptively in response to detected blood pressure elevations. Similarly, piezoelectric or mechanoresponsive nanocomposites could convert the arterial pulse or pressure changes into physical or chemical stimuli to trigger on-demand drug release. These approaches illustrate how nanomaterials could actively regulate blood pressure by linking sensing and therapeutic functions, moving beyond passive monitoring toward adaptive, spatiotemporally controlled interventions.

Thus, current nanomaterial-based systems provide not only high-precision monitoring but also a promising platform for developing active regulation strategies in the adjunctive stage of ischemic stroke care, potentially mitigating reperfusion-related complications and improving patient outcomes.

#### 2.4.2 Therapeutic hypothermia

In ischemic stroke, elevated brain temperature exacerbates oxidative stress and inflammatory responses, accelerating neuronal apoptosis and the expansion of the infarct core (Mezuki et al., 2024). TH has been widely investigated as an adjunctive strategy to mitigate secondary brain injury following reperfusion. By lowering brain temperature, TH can decrease metabolic demand, stabilize the BBB, and inhibit inflammatory cascades (Sun et al., 2024). Clinically, TH can be induced through systemic cooling (e.g., cold saline infusion, ice packs), pharmacologic agents, or selective brain hypothermia (SBH) using methods such as carotid cold saline perfusion or ice cap cooling (Mayer et al., 2004; Liu et al., 2016; Lee et al., 2016; Clark and Colbourne, 2007; Poli et al., 2014; Kurisu et al., 2016a; Kurisu et al., 2016b; Esposito et al., 2014; Chen et al., 2016). However, these conventional approaches face multiple limitations, including poor heat transfer efficiency, imprecise temperature control, and systemic side effects like shivering and hypotension. In addition, most temperature monitoring relies on invasive probes, restricting its practicality for real-time feedback in stroke care.

To address these limitations, nanomaterial-based platforms have been developed for non-invasive, localized, and real-time temperature monitoring, enhancing both precision and safety of hypothermia interventions Lanthanide luminescent nanoparticles (LLNs) are particularly promising in this context due to their high tissue penetration and temperature-responsive emission properties (Li et al., 2023). These nanoparticles typically adopt a core shell architecture consisting of a NaNdF_4_:Yb core, a CaF_2_ interlayer, and a NaNdF_4_:Y shell, which enables strong near-infrared luminescence and temperature-dependent signal output. Under 808 nm near-infrared excitation, LLNs emit at distinct wavelengths whose intensity ratio can be used to calculate brain temperature in real time with high spatial resolution. After intravenous administration, LLNs can cross the compromised BBB and preferentially accumulate in the infarcted hemisphere, enabling localized thermometry for guiding therapeutic cooling. Experimental data further indicate a positive correlation between infarct temperature and inflammatory cytokine levels such as TNF-α, suggesting that precise temperature control may contribute to mitigation of neuroinflammation. Compared with traditional thermometry methods, LLNs offer a minimally invasive, infarct-targeted, and continuously monitorable solution, potentially allowing dynamic adjustment of hypothermia protocols to prevent both overcooling and undercooling.

Although LLNs currently provide passive high-resolution thermometry, these platforms lay the groundwork for future active thermal regulation. For instance, LLNs could be integrated with thermo-responsive or phase-change nanomaterials to create feedback-guided, self-regulating hypothermia systems. In such systems, real-time temperature readouts could trigger localized photothermal modulation or controlled release of cooling agents, enabling active adjustment of brain temperature in response to evolving ischemic conditions. These multifunctional nanosystems represent a promising strategy for combining monitoring with active intervention, potentially enhancing the precision and neuroprotective efficacy of TH in the adjunctive stage of ischemic stroke.

#### 2.4.3 Oxygen therapy

In the adjunctive stage of ischemic stroke, oxygen therapy serves as a supportive strategy to mitigate secondary brain injury following revascularization (Satyarthee, 2019; Hu et al., 2023). By improving oxygen availability in the ischemic penumbra, oxygen therapy aims to limit hypoxia-induced oxidative stress, mitochondrial dysfunction, and neuronal apoptosis. Traditionally, two main approaches have been employed in clinical and preclinical settings: normobaric oxygen therapy (NBO) and hyperbaric oxygen therapy (HBOT) (Hu et al., 2023; Yang et al., 2024). NBO delivers high-concentration oxygen under normal atmospheric pressure (Ding et al., 2019), while HBOT administers pure oxygen at elevated pressures to enhance oxygen diffusion into hypoperfused areas (Bosco et al., 2025). Although these methods have demonstrated neuroprotective effects in some animal models, clinical outcomes remain inconsistent. Factors such as limited targeting capability, potential for oxygen toxicity, and reliance on specialized equipment have hindered their translational success and therapeutic consistency in stroke management (Gupta and Somasundaram, 2023).

To overcome these limitations, nanotechnology-based oxygen delivery platforms have emerged to enable localized, sustained, and stimulus-responsive oxygen supplementation. One representative example is fluorocarbon-functionalized hollow mesoporous organosilica nanoparticles (FHMONs) (Chen et al., 2017).

Importantly, this nanoplatform was originally developed and validated in tumor hypoxia models rather than ischemic stroke. Therefore, it is discussed here primarily as a translationally informative design rather than a stroke-validated therapeutic strategy. In FHMONs, fluorocarbon chains known for their high oxygen solubility are covalently integrated into a rigid organosilica framework, minimizing premature oxygen leakage and prolonging circulation time compared with conventional perfluorocarbon emulsions.

In preclinical tumor models, FHMONs demonstrated the ability to accumulate in hypoxic tissues and release oxygen in a controlled, ultrasound-triggered manner, enabling on-demand delivery with spatiotemporal precision. This design is particularly relevant to ischemic stroke, where tissue oxygenation after vascular recanalization is often spatially heterogeneous, and excessive systemic oxygen supplementation may exacerbate oxidative stress. By providing localized and externally triggered oxygen release, FHMONs may theoretically improve regional oxygen availability while limiting off-target hyperoxia compared with conventional oxygen therapy.

Nevertheless, direct evidence in ischemic stroke models is still lacking. Future studies should evaluate whether FHMONs can effectively accumulate in ischemic or peri-infarct brain regions, improve tissue oxygenation without exacerbating ROS-mediated injury, reduce infarct volume, and enhance neurological recovery in experimental stroke models.

Beyond oxygen delivery, nanotechnology is also being explored for real-time sensing and regulation of key physiological parameters in the adjunctive stage. Table 6 summarizes representative nano-sensing and regulatory platforms that could enhance adjunctive care in ischemic stroke, including real-time monitoring of blood pressure, brain temperature, and targeted oxygen delivery.

**TABLE 6:** Nano-sensing and regulatory platforms in the adjunctive stage.

Platform	Function	Target	Strategy	Key finding	Stage
Graphene-based nanosensor	Monitoring	Blood pressure	Flexible piezoresistive sensing	Enables continuous and responsive BP monitoring	Preclinical
Lanthanide luminescent nanoparticles	Monitoring	Brain temperature	NIR nanothermometry	Allows real-time infarct-region temperature tracking	Preclinical
FHMON oxygen-delivery nanoparticles	Regulation	Tissue oxygenation	Ultrasound-triggered oxygen release	Provides controllable oxygen supplementation in hypoxic tissue	Conceptual/Preclinical
Emerging multifunctional nanosystems	Monitoring and regulation	Hemodynamic and metabolic status	Integrated sensing-delivery design	Proposed to enable closed-loop adjunctive management	Conceptual

Adjunctive stroke management further highlights the importance of spatiotemporal control. Unlike conventional supportive therapies that rely on intermittent monitoring and static interventions, nanotechnology enables continuous sensing and responsive regulation of physiological parameters such as blood pressure, temperature, and oxygen levels. These advances provide the foundation for real-time feedback-guided interventions and suggest a transition toward more dynamic and adaptive stroke management strategies.

Nevertheless, most currently reported nanosystems focus primarily on either sensing or therapeutic modulation. Achieving seamless integration between these functions therefore remains a key challenge for the clinical translation of adjunctive nanomedicine. Although the four stages described above can be conceptually separated according to their dominant therapeutic objectives, ischemic stroke progression is inherently dynamic, and the underlying pathological mechanisms often overlap across stages. Therefore, understanding how therapeutic strategies transition and coordinate between these stages is essential for achieving continuous and effective stroke management.

### 2.5 Stage connection and dynamic regulation

Although prevention, neuroprotection, revascularization, and adjunctive care can be conceptually divided into distinct therapeutic stages, ischemic stroke itself represents a dynamically evolving pathological process. Vascular risk factor accumulation, thrombus formation, ischemic injury, reperfusion-related damage, and subsequent tissue repair occur along a continuous temporal spectrum rather than in strictly separated phases. Consequently, effective stroke therapy should not rely solely on isolated stage-specific interventions but should also consider how therapeutic strategies can be coordinated across stages to ensure continuous and adaptive treatment.

From a clinical perspective, the prevention stage establishes the physiological baseline that influences stroke susceptibility and the severity of subsequent ischemic injury. Effective management of hypertension, dyslipidemia, platelet activation, and coagulation abnormalities not only reduces stroke incidence but may also alleviate vascular vulnerability once ischemia occurs. Following stroke onset, the neuroprotection stage functions as a critical bridge before reperfusion therapies by preserving the ischemic penumbra and delaying irreversible neuronal injury. This stage helps extend the therapeutic window for revascularization strategies such as thrombolysis or mechanical thrombectomy. After successful recanalization, however, the restoration of cerebral blood flow may trigger additional pathological processes, including oxidative stress amplification, inflammatory responses, blood pressure fluctuations, and blood-brain barrier instability. Therefore, the adjunctive stage should be viewed as a continuation of acute intervention, aiming to stabilize systemic and cerebral physiological parameters and create favorable conditions for neurological recovery.

Nanomedicine provides a promising platform for enabling dynamic therapeutic regulation across these interconnected stages. Temporally, nanocarriers can be engineered to achieve sustained drug release during long-term prevention, rapid brain delivery during the neuroprotection stage, thrombus-responsive activation during revascularization, and feedback-guided physiological regulation during adjunctive care. Spatially, therapeutic targets may evolve from systemic vascular risk factors and circulating coagulation components to the ischemic penumbra, inflammatory cells, endothelial barriers, and subcellular organelles such as mitochondria. Moreover, emerging multifunctional nanosystems integrating biosensing, targeted delivery, and stimulus-responsive actuation may enable closed-loop therapeutic strategies that adapt interventions according to real-time physiological signals. Such dynamic regulation highlights the potential of nanomedicine to support continuous therapeutic intervention throughout the entire course of ischemic stroke management, from risk control to post-reperfusion recovery.

## 3 From bench to bedside: translational challenges and perspectives

It should be noted that not all nanoplatforms discussed in this review have been directly validated in ischemic stroke models. Some systems are included because they address pathophysiological demands highly relevant to stroke, such as targeted anticoagulation, localized oxygen delivery, or stimulus-responsive drug release. These platforms therefore provide valuable translational insights even when direct evidence in stroke models is still limited. Future studies should prioritize validation in cerebrovascular disease models to fully establish their therapeutic potential.

Despite encouraging preclinical results, the clinical translation of nanotechnology for ischemic stroke remains constrained by several practical and regulatory barriers. These challenges extend beyond proof-of-concept efficacy and involve long-term biosafety evaluation, scalable manufacturing, regulatory approval, and integration into existing clinical workflows.

Long-term biosafety remains a central concern. Nanomaterials may accumulate in off-target organs or elicit unintended immune responses, particularly when repeated administration is required. The development of biodegradable materials, together with standardized long-term toxicity assessment strategies, will be essential for ensuring clinical safety.

Scalable manufacturing represents another major barrier. Many nanoplatforms rely on complex multistep synthesis processes that are difficult to reproduce at large scale, leading to batch-to-batch variability. Establishing GMP-compatible and modular production systems will be critical for ensuring consistency and facilitating industrial translation.

Regulatory pathways for nanomedicine remain insufficiently defined. The lack of nano-specific evaluation standards may delay clinical approval and increase development costs. Early engagement with regulatory agencies and the establishment of harmonized assessment frameworks could help accelerate translation.

Targeting reproducibility is also challenged by interpatient variability, particularly in blood-brain barrier status and post-reperfusion microenvironmental differences. Precision-oriented and stimuli-responsive nanosystems may help improve therapeutic consistency across heterogeneous patient populations.

In addition, cost-effectiveness and clinical integration must be considered. Simplified material design, scalable fabrication strategies, and compatibility with existing stroke treatment workflows will be essential for practical implementation.

The major translational barriers and corresponding mitigation strategies discussed above are summarized in Table 7.

**TABLE 7:** Translational challenges and potential solutions.

Major challenge	Key issue	Potential solution
Long-term biosafety	Biodistribution uncertainty and accumulation	Biodegradable materials, long-term toxicity studies
Manufacturing scale-up	Batch variability and complexity	GMP-compatible modular production platforms
Regulatory pathway	Lack of nano-specific approval processes	Early regulatory engagement, harmonized standards
Targeting variability	Patient heterogeneity and variability in BBB status	Stimuli-responsive nanomedicines, patient stratification
Cost-effectiveness	High production costs and complexity	Scalable, cost-effective materials and simplified designs
Clinical integration	Compatibility with existing clinical workflows	Imaging-guided and interoperable systems

## 4 Emerging future directions of nanomedicine

Beyond the translational challenges discussed above, several emerging scientific directions may further expand the therapeutic potential of nanomedicine for ischemic stroke. Recent studies suggest that future advances may arise from three major areas: system-level regulation through the gut-brain axis, gene therapy-enabled nanomedicine, and intelligent nanosystems capable of integrating sensing and therapeutic functions.

Growing evidence indicates that ischemic stroke is associated with systemic immune responses and alterations in gut microbiota composition, suggesting that modulation of the gut-brain axis may provide an indirect strategy for regulating neuroinflammation and promoting neurological recovery. In parallel, nanocarriers capable of delivering gene-editing tools such as CRISPR/Cas systems, siRNA, or mRNA across the blood-brain barrier may enable precise regulation of molecular pathways involved in oxidative stress, inflammation, and neuronal regeneration. Furthermore, the integration of sensing and therapeutic capabilities within nanoplatforms may enable closed-loop therapeutic systems that dynamically respond to pathological signals such as oxidative stress or inflammatory mediators.

**FIGURE 10:**
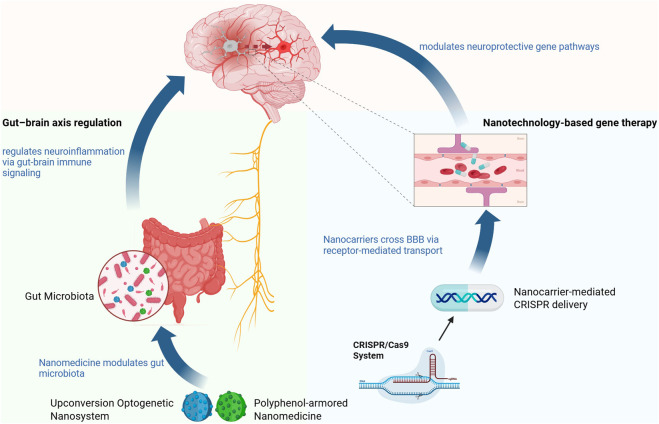
Emerging paradigms of nanomedicine for ischemic stroke. Schematic illustration of emerging research directions in nanomedicine that may expand future therapeutic strategies for ischemic stroke. On the left, nanomedicine-mediated modulation of gut microbiota may regulate neuroinflammation through gut-brain immune signaling, highlighting the potential of microbiota-targeted nanosystems such as polyphenol-armored nanomedicine and upconversion optogenetic platforms. On the right, nanotechnology-enabled gene delivery systems may allow nanocarriers to transport CRISPR/Cas-based gene-editing tools across the blood-brain barrier (BBB), enabling targeted modulation of neuroprotective gene pathways. Together, these emerging paradigms illustrate how nanomedicine may provide new opportunities for system-level regulation and molecular-level intervention in future stroke management.

Together, these emerging directions highlight the potential for next-generation nanomedicine systems capable of achieving adaptive and spatiotemporally controlled stroke therapy. Representative emerging research directions, including gut-brain axis modulation and nanotechnology-enabled gene delivery, are schematically illustrated in Figure 10. Among these emerging directions, intelligent and closed-loop nanosystems represent one of the most promising approaches for enabling real-time adaptive intervention during stroke progression.

### 4.1 Intelligent and closed-loop nanomedicine systems

Future nanomedicine systems may evolve toward intelligent and closed-loop therapeutic platforms capable of enabling precise, real-time modulation of key physiological parameters such as blood pressure, brain temperature, and oxygenation. However, despite substantial progress, current research remains disproportionately focused on long-term blood pressure management and sustained drug delivery systems, which do not adequately address the acute, rapidly evolving challenges of the revascularization stage. Similarly, although nanomaterials have greatly improved temperature sensing accuracy, most existing platforms remain passive in nature and lack the ability to initiate controlled hypothermia. Recent innovations in oxygen delivery, such as FHMONs, offer promising strategies for localized, stimulus-responsive oxygen supplementation. Nevertheless, their application in stroke remains at the conceptual stage, with critical issues such as targeting efficiency, biosafety, and therapeutic consistency yet to be fully resolved. To overcome these limitations, future research should focus on the development of artificial intelligence (AI), multifunctional nanoplatforms capable of integrating monitoring and intervention in a closed-loop, responsive manner. The convergence of nanotechnology with wearable biosensors and AI-driven analytics holds great potential for establishing a new standard of personalized adjunctive care. Such systems could dynamically adjust therapeutic strategies in response to evolving cerebral conditions, thereby stabilizing systemic parameters, reducing secondary injury, and ultimately improving functional recovery in the post-revascularization period.

### 4.2 Gut-brain axis modulation

Ischemic stroke is not a purely localized event within the brain. Increasing evidence highlights its systemic nature, implicating complex interactions between the brain, immune system, and gut microbiota (Mathias et al., 2024; Benakis and Liesz, 2022). Recent studies suggest that ischemic stroke triggers not only a central nervous system response but also alters peripheral immune and metabolic pathways, with the gut-brain axis playing a central role in modulating stroke outcomes.

The immune response following ischemic stroke is intricately connected to brain injury and recovery. Activation of immune cells, such as microglia and astrocytes, in the ischemic brain results in the release of inflammatory cytokines that further exacerbate neuronal damage and impede recovery (Xu et al., 2024). In parallel, the gut microbiota has been shown to influence these immune responses through the gut-brain axis, exacerbating systemic inflammation and affecting brain repair mechanisms (Wang et al., 2025; Xian et al., 2024; Yamashiro et al., 2021). The modulation of gut microbiota composition may, therefore, represent an innovative strategy to regulate systemic immune responses and neuroinflammation, ultimately influencing stroke progression and recovery.

Nanomedicine presents a novel opportunity to modulate the gut-brain axis, thereby indirectly impacting stroke progression. Nanocarriers can be engineered to selectively deliver microbiota-modulating agents, such as prebiotics, probiotics, or microbiota-derived metabolites, to reshape gut microbial communities after ischemic stroke. Recent studies have demonstrated the potential of orally administered polyphenol-armored nanomedicines in treating colitis by modulating the gut microbiota and its interaction with the brain. This nanomedicine alleviates intestinal inflammation, reduces systemic inflammation, and improves emotional and cognitive disorders associated with gut-brain interactions (He et al., 2023). These findings highlight the potential of nanomedicines to influence systemic immune responses, demonstrating a new therapeutic avenue for managing stroke-induced inflammation and improving recovery.

In a similar vein, Pan et al. developed an upconversion optogenetic micro-nano system that allows for the precise delivery and expression of light-sensitive probiotics within the gut. This system enables targeted regulation of the gut-brain axis, with potential applications in treating diseases such as Parkinson’s disease, anxiety, and vagus nerve-related conditions (Pan et al., 2022). Though not directly related to ischemic stroke, these studies underscore the transformative potential of nanomedicine in modulating complex intersystem interactions, such as the gut-brain axis, to achieve therapeutic benefits. The ability to leverage such technologies for stroke treatment is especially promising in addressing secondary complications and influencing key pathological mechanisms such as neuroinflammation, cerebral perfusion, and neuroprotection.

From a mechanistic perspective, several hypotheses can be proposed regarding the role of nanomedicines in gut-brain modulation following ischemic stroke. A potential strategy involves the use of nanocarriers to selectively deliver microbiota-modulating agents, such as short-chain fatty acids (SCFAs) or secondary bile acids, which are known to influence immune responses and neuroinflammation. These metabolites can regulate systemic immune responses and attenuate microglia-mediated neuroinflammation in the ischemic brain. For example, SCFAs like butyrate have been shown to reduce pro-inflammatory cytokine production and enhance the blood-brain barrier integrity, which could aid in mitigating post-stroke neuroinflammation (Fock and Parnova, 2023).

Nanomedicines may also provide targeted delivery of engineered probiotics or prebiotics to reshape the gut microbiota composition. By influencing the gut microbiome’s metabolic activity, such as increasing the production of anti-inflammatory metabolites, these nanosystems could provide an additional layer of therapeutic regulation beyond direct neurovascular targeting. This gut-brain axis modulation could complement existing stroke therapies by targeting systemic immune responses, thus offering a holistic approach to stroke recovery.

In summary, nanomedicines targeting the gut-brain axis hold significant promise in ischemic stroke management. By modulating gut microbial metabolism, these therapies could reduce neuroinflammation, improve cerebral perfusion, and enhance neuroprotection and repair. While current research is still in its infancy, the integration of nanotechnology with microbiota-based therapies presents an exciting Frontier in stroke treatment, with the potential for transformative advances in personalized medicine.

### 4.3 Gene therapy nanomedicine

Gene therapy offers a direct approach to intervene in ischemic injury by regulating disease-driving genes and pathways at both the transcriptional and post-transcriptional levels. However, its clinical translation faces significant challenges, particularly in achieving safe and selective delivery to the central nervous system (CNS), especially across the BBB. Despite the tremendous therapeutic potential of gene-editing technologies such as CRISPR/Cas9, their clinical application is still hindered by issues such as immunogenicity, off-target effects, and the narrow therapeutic time window following stroke (Wang et al., 2024a).

Nanotechnology has emerged as a promising solution for gene therapy delivery, with LNPs being one of the most promising non-viral vectors for delivering CRISPR/Cas9 gene-editing tools. LNPs have shown excellent stability, biocompatibility, and the ability to cross the BBB, effectively delivering CRISPR systems to target cells. By carefully designing LNPs, researchers can incorporate charge-modulating groups on the particle surface to efficiently encapsulate mRNA, DNA, or other genetic materials while minimizing systemic side effects (Jiang et al., 2026). For example, lipid nanoparticles protect nucleic acids from enzymatic degradation, enhance cellular uptake, and facilitate endosomal escape, collectively ensuring efficient and targeted gene delivery to neural tissues. ONPATTRO, an FDA-approved LNP-siRNA drug, demonstrates the potential of LNP-based delivery in clinical applications, providing a reference for the delivery of CRISPR gene-editing tools (Lim et al., 2022).

Recent preclinical studies have begun to demonstrate the feasibility of nanotechnology-enabled CRISPR-based gene modulation in ischemic stroke models. For example, Ryu et al. developed an intranasally delivered nanoparticle platform encapsulating a protein-based CRISPR/dCas9 transcriptional activator targeting the neuroprotective gene Sirt1(Ryu et al., 2024). In this system, dCas9-VP64 complexes were packaged within calcium phosphate nanoparticles and modified with β-hydroxybutyrate-conjugated polymers to facilitate efficient nose-to-brain transport. In a permanent middle cerebral artery occlusion (pMCAO) mouse model, this nanosystem successfully enhanced Sirt1 expression in the ischemic brain, reduced cerebral edema, and improved survival outcomes. These findings provide a proof-of-concept example demonstrating that nanoparticle-assisted CRISPR systems can modulate neuroprotective gene pathways in stroke-relevant conditions, highlighting their potential for early-stage translational development in ischemic stroke therapy.

However, despite the promising potential of LNPs for gene delivery, gene therapy for ischemic stroke still faces several challenges. The pathophysiology following stroke is complex and progresses rapidly, with oxidative stress and neuroinflammation exacerbating brain injury after reperfusion, making timely treatment difficult (Wang et al., 2024a). In addition, the permeability of the BBB changes dynamically during stroke progression, which further complicates the delivery efficiency of gene therapeutics. Therefore, the key issue remains how to deliver gene therapies at precise spatial and temporal points. One of the key advantages of nanomedicine is its ability to provide spatiotemporal control. By utilizing stimuli-responsive features, such as pH-sensitive or ultrasound-triggered release mechanisms, nanoparticle systems can ensure gene therapies are released precisely in the ischemic region, thus avoiding systemic distribution and enhancing therapeutic effects, aligning with stage-specific intervention strategies for ischemic stroke.

Looking forward, gene therapy has the potential to not only repair damaged DNA but also modulate gene expression at the epigenetic level, further improving neuroprotection and recovery after stroke. For instance, using CRISPR to regulate genes associated with neuroprotection, mitochondrial function, and inflammation could provide new therapeutic avenues for long-term recovery post-stroke. Future nanoplatforms integrating gene delivery with other nanomedicine strategies—such as antioxidant nanozymes or anti-inflammatory nanocarriers—may enable multi-target and stage-adaptive interventions that better address the complex pathological cascade of ischemic stroke. The integration of gene therapy with other neuroprotective strategies, such as antioxidant and anti-inflammatory treatments, may significantly enhance treatment outcomes.

While gene therapy holds immense potential for treating ischemic stroke, its clinical application will depend on overcoming challenges such as efficient and targeted delivery, optimizing nanoparticle formulations, and ensuring biosafety and scalability. Advances in nanotechnology-driven spatiotemporal control are expected to play a key role in enabling precise and stage-specific gene therapy for ischemic stroke in the future.

## 5 Conclusion and prospects

This review highlights how nanomedicine-based strategies can address therapeutic challenges across the key stages of ischemic stroke management, including prevention, neuroprotection, revascularization, and adjunctive care. By integrating advances in nanotechnology with stage-specific therapeutic needs, nanomedicine provides new opportunities to overcome several limitations associated with conventional stroke treatments.

Nanotechnology offers unique advantages for improving drug delivery efficiency, enhancing BBB penetration, and enabling targeted therapeutic intervention in ischemic brain regions. Through rational nanocarrier design, therapeutic agents can achieve improved bioavailability, optimized pharmacokinetics, and reduced systemic toxicity. In addition, stimuli-responsive nanoplatforms allow controlled drug release in response to pathological cues, enabling therapeutic activity to better match the dynamic progression of ischemic injury.

Importantly, nanomedicine also provides opportunities to simultaneously modulate multiple pathological mechanisms involved in ischemic stroke, including oxidative stress, excitotoxicity, inflammatory responses, and mitochondrial dysfunction. Such multi-target and multi-mechanism strategies may enhance neuroprotection and support the repair of damaged neural tissue, thereby improving neurological recovery and long-term functional outcomes.

Despite these promising advances, several challenges remain for the clinical translation of nanomedicine in ischemic stroke. Issues such as long-term biosafety, large-scale manufacturing, regulatory approval, and integration with established clinical treatment paradigms must be carefully addressed before widespread clinical application can be achieved.

Looking forward, the development of next-generation nanoplatforms capable of integrating targeted delivery, controlled drug release, and real-time therapeutic regulation may further improve treatment precision. Such intelligent and multifunctional nanosystems hold considerable promise for enabling more adaptive and personalized therapeutic strategies in ischemic stroke management.

Overall, nanomedicine represents a versatile platform with significant potential to overcome key limitations of conventional stroke therapies and to advance the development of more precise and effective treatment paradigms for ischemic stroke.
